# Role of Carbonyl Compounds for *N*-Nitrosamine Formation during Nitrosation: Kinetics and Mechanisms

**DOI:** 10.1021/acs.est.3c07461

**Published:** 2024-03-01

**Authors:** Yishuai Pan, Florian Breider, Benjamin Barrios, Daisuke Minakata, Huiping Deng, Urs von Gunten

**Affiliations:** † School of Architecture, Civil and Environmental Engineering (ENAC), Ecole Polytechnique Fédérale Lausanne (EPFL), CH-1015 Lausanne, Switzerland; ‡ Key Laboratory of Yangtze River Water Environment, Ministry of Education, Shanghai Institute of Pollution Control and Ecological Security, College of Environmental Science and Engineering, Tongji University, Shanghai 20092, China; § Department of Civil, Environmental and Geospatial Engineering, Michigan Technological University, 1400 Townsend Drive, Houghton, Michigan 49931, United States; ∥ Eawag, Swiss Federal Institute of Aquatic Science and Technology, CH-8600 Dübendorf, Switzerland

**Keywords:** *N*-nitrosamines, carbonyl compounds, iminium ions, carbinolamines, nitrosation, water and wastewater treatment

## Abstract

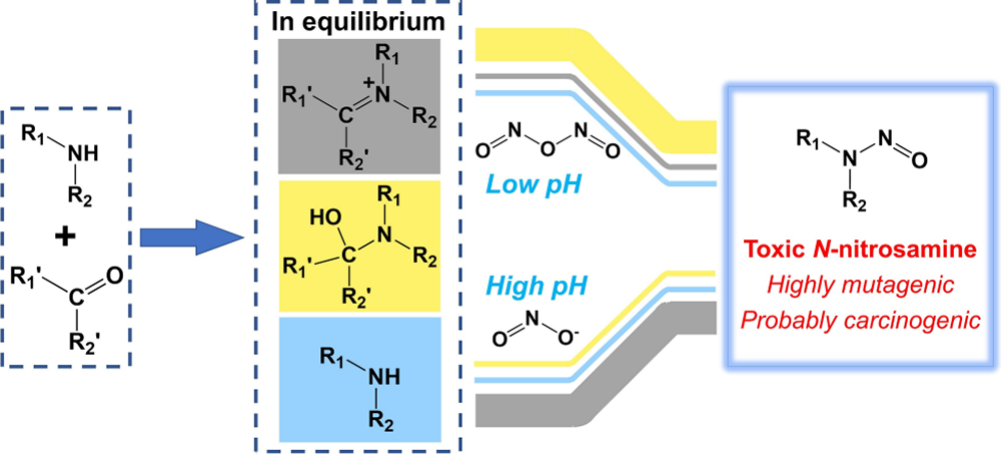

*N*-Nitrosamines are potential human carcinogens frequently detected in natural and engineered aquatic systems. This study sheds light on the role of carbonyl compounds in the formation of *N*-nitrosamines by nitrosation of five secondary amines via different pathways. The results showed that compared to a control system, the presence of formaldehyde enhances the formation of *N*-nitrosamines by a factor of 5–152 at pH 7, depending on the structure of the secondary amines. Acetaldehyde showed a slight enhancement effect on *N*-nitrosamine formation, while acetone and benzaldehyde did not promote nitrosation reactions. For neutral and basic conditions, the iminium ion was the dominant intermediate for *N*-nitrosamine formation, while carbinolamine became the major contributor under acidic conditions. Negative free energy changes (<−19 kcal mol^–1^) and relatively low activation energies (<18 kcal mol^–1^) of the reactions of secondary amines with N_2_O_3_, iminium ions with nitrite and carbinolamines with N_2_O_3_ from quantum chemical computations further support the proposed reaction pathways. This highlights the roles of the iminium ion and carbinolamine in the formation of *N*-nitrosamines during nitrosation in the presence of carbonyl compounds, especially in the context of industrial wastewater.

## Abstract 2

In the presence of carbonyl compounds, nitrosation of secondary amines to *N*-nitrosamines can be significantly enhanced via carbinolamine and iminium ion pathways.

## Introduction

*N*-Nitrosamines are potential human carcinogens, which can be present in food and consumables, but are also formed as disinfection byproducts during oxidative water and wastewater treatment by reactions of chlorine, chloramines, and ozone with nitrogen-containing compounds.^1−3^ The formation and mitigation of *N*-nitrosamines has been extensively investigated in the last decades in engineered aquatic systems.^4−7^ Six *N*-nitrosamines that potentially occur in aquatic systems are currently on the US EPA Contaminant Candidate List 5 (CCL5) in the group “disinfection byproducts”.^8^ For example, *N*-nitrosodimethylamine (NDMA), the most commonly detected *N*-nitrosamine in drinking water and wastewater, poses a 10^–6^ cancer risk at a concentration of 0.7 ng/L.^1^ In contrast, the concentration of chloroform for the same cancer risk is 6 μg/L, which corresponds to about a 1000-fold lower toxicity than NDMA.^9^ Drinking water guidelines for NDMA were established to be 100 and 40 ng/L by the World Health Organization (WHO)^10^ and the Canadian health authorities,^11^ respectively. A notification level of 10 ng/L was set for 3 *N*-nitrosamines by California’s Department of Public Health (CDPH).^12^

**Scheme 1:**
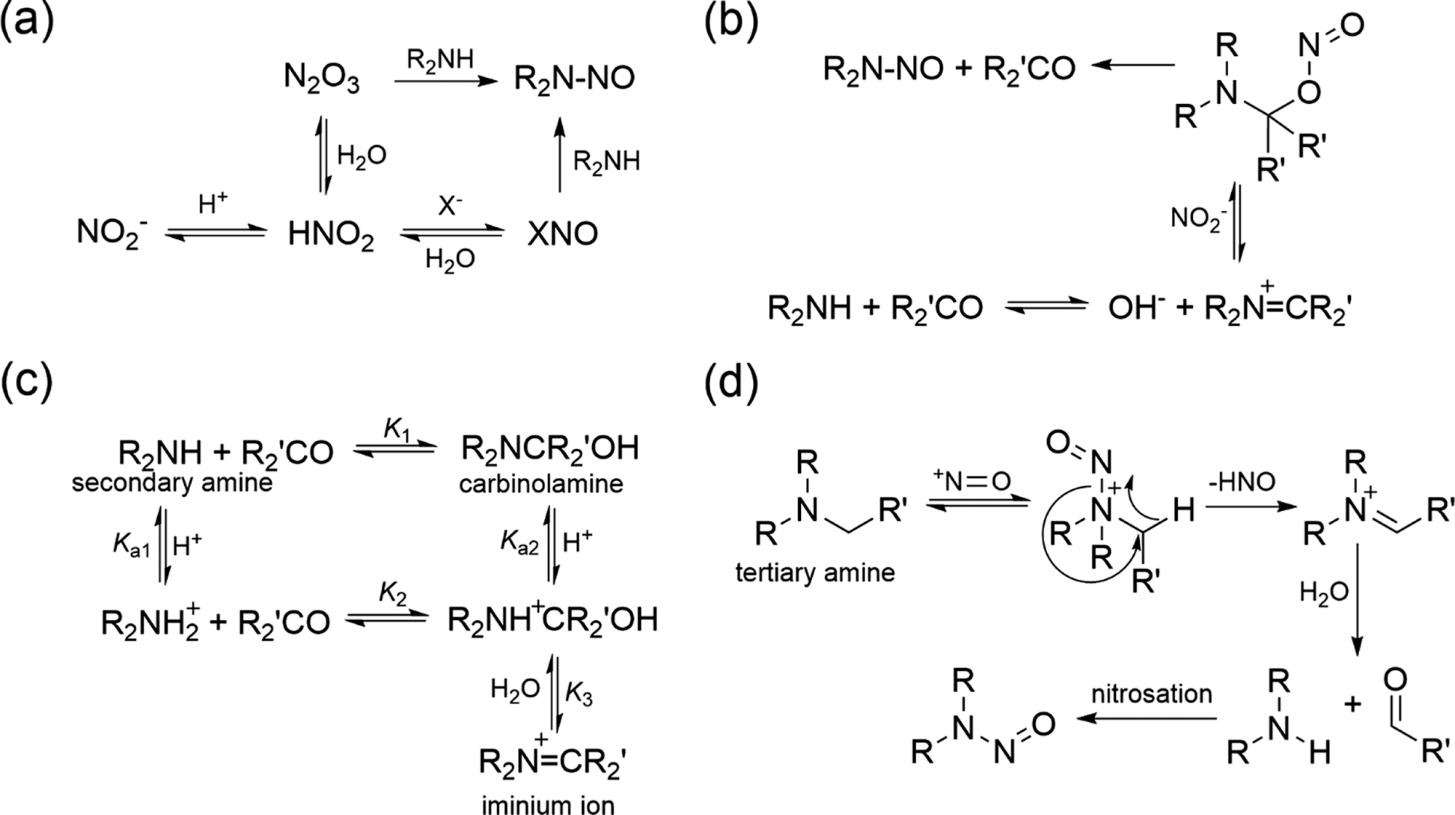
Reactions Related to the Nitrosation Process *N*-Nitrosamine formation in (a) the absence^28^ and (b) the presence^30^ of carbonyl compounds. (c) Equilibria for the reactions between secondary amines and carbonyl compounds,^33^ and (d) *N*-nitrosamine formation from simple tertiary amines.^34^

Even though in water treatment, oxidation processes and especially chloramination are the main drivers for *N*-nitrosamine formation,^2,13^ nitrosation processes may become an important pathway when secondary amines, nitrite, and carbonyl compounds are present simultaneously in engineered aquatic systems (e.g., in sewer systems or wastewater treatment plants where municipal and industrial wastewaters are mixed).^14,15^ They are common wastewater components and their concentrations can reach up to tens of mg/L.^16−21^ One case in point, which may be a consequence of the reactions of these compounds, is a survey on *N*-nitrosamines in industrial wastewaters in Switzerland, in which *N*-nitrosamine concentrations of almost 1 mg/L were detected.^22^ Nitrosation reactions can also occur in water systems receiving effluents from industrial cooling or hydraulic fracturing processes,^23,24^ amine-based CO_2_ capture systems,^25^ food processing^26^ and GAC-based methods for *N*-nitrosamine analysis.^27^ Nitrite itself is a weak nitrosating agent, however, it enhances *N*-nitrosamine formation at low pH (<6) by production of strong nitrosating agents such as dinitrogen trioxide (N_2_O_3_) and nitrosyl halides (XNO, X = Cl^–^/Br^–^) (eqs 1–3).^28^ The corresponding second-order rate constants for the reactions with secondary amines are in the order of 10^6^–10^9^ M^–1^ s^–1^ (eqs 4, 5 and Scheme 1a).^29^
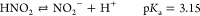
1
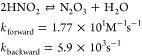
2
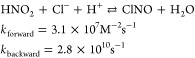
3
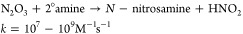
4
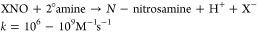
5

The nitrosation process is most favorable under acidic conditions (Scheme 1a). Nevertheless, under neutral and basic conditions, an alternative pathway for *N*-nitrosamine formation proceeds via formaldehyde and chloral catalysts (Scheme 1b).^30^ The proposed *N*-nitrosamine formation mechanism in the presence of carbonyl compounds includes an initial reaction between secondary amines and carbonyl compounds (Scheme 1c).^31^ This reaction yields a carbinolamine, which easily releases a hydroxyl group to yield an iminium ion. Iminium ions are known to have high reactivity with nucleophiles such as nitrite, which yields *N*-nitrosamines and the parent carbonyl compounds (Scheme 1b).^30^ However, iminium ions are unstable and hydrolyze rapidly in aqueous solutions. One case in point is H_2_C = N^+^(CH_3_)CH_2_CF_3_, an aliphatic iminium ion which has a hydrolysis rate constant of (1.8 ± 0.1) × 10^7^ s^–1^ and a lifetime of (5.5 ± 0.3) × 10^–8^ s.^32^ Therefore, the transient concentrations of iminium ions in aqueous solutions are extremely low and may not be relevant to the formation of significant concentrations of *N*-nitrosamine in aquatic systems.

Carbinolamines, the precursors of iminium ions, are always in equilibrium with secondary amines and iminium ions in aqueous solution (Scheme 1c). They are tertiary amine-type compounds and may also be involved in the nitrosation process with formation of the corresponding *N*-nitrosamines.^35,36^ Previous studies suggest that tertiary amines may first undergo a dealkylation process to yield a secondary amine, which can then be nitrosated to *N*-nitrosamine (Scheme 1d).^34^ In their neutral form, tertiary amines also have a lone electron pair on the amine nitrogen, which may attack N_2_O_3_, similar to the case of secondary amines. Meanwhile, the dissociation constant (*K*_a_) of carbinolamines was reported to be 2–3 orders of magnitude lower than that of the parent secondary amines.^33^ This means that a higher fraction of carbinolamine is in its neutral form at the same pH value, which can contribute to the formation of the corresponding *N*-nitrosamine. To assess the extent of nitrosation reactions in the presence of carbonyl compounds, kinetic and mechanistic information about *N*-nitrosamine formation is needed.

This study investigated the *N*-nitrosamine formation by nitrosation in nitrite- and carbonyl compound-containing solutions. The objectives of this study were (1) to elucidate the formation kinetics of *N*-nitrosamines from secondary amines in the pH-range 4–8 and (2) to investigate the mechanisms of *N*-nitrosamine formation including different potential precursors such as secondary amines, carbinolamines, and iminium ions. In addition, quantum chemical computations and kinetic simulations were performed to support the proposed mechanism, allowing a comprehensive understanding of the fate of different reactive species during nitrosation. Finally, nitrosation processes were assessed for different water qualities by kinetic modeling.

## Materials and Methods

### Standards and Reagents

Dimethylamine (DMA), diethylamine (DEA), sulfamic acid, formaldehyde (HCHO, 37 wt % in H_2_O containing 10–15% methanol as a stabilizer), acetone, and benzaldehyde were obtained from Sigma-Aldrich, pyrrolidine (PYR), morpholine (MOR), sodium nitrite, and acetaldehyde from Merck, and *N*-methylethylamine (MEA) from Tokyo Chemical Industry (TCI) Co., Ltd. The US-EPA 8270 *N*-nitrosamines mix (2000 μg/mL each component in methanol), containing *N*-nitrosodibutylamine (NDBA), *N*-nitrosodiethylamine (NDEA), *N*-nitrosodimethylamine (NDMA), *N*-nitrosodiphenylamine (NDPhA), *N*-nitrosodi-n-propylamine (NDPA), *N*-nitrosomethylethylamine (NMEA), *N*-nitrosomorpholine (NMOR), *N*-nitrosopiperidine (NPIP), and *N*-nitrosopyrrolidine (NPYR), was obtained from Sigma-Aldrich. All chemicals were of analytical grade and used as received without further purification.

### Analytical Methods

The *N*-nitrosamine concentrations were measured using an Agilent 1290 HPLC system coupled with a diode array detector (DAD) at 230 nm and a C_18_ column (Acclaim Polar Advantage II, 120 Å, 5 μm, 4.6 mm × 150 mm, Thermo Scientific).^37^ The mobile phase consisted of HPLC-grade acetonitrile (phase A) and 10 mM phosphoric acid (phase B) at a flow rate of 0.8 mL/min. The gradient elution procedure started from 98% A for the first 6 min, then decreased to 20% in the following 12 min, and was kept for 5 min; finally, it was increased to 98% A again within 5 min and maintained for 4 min.

### Kinetic Experiments

All kinetic experiments were conducted in 10 mL amber borosilicate bottles containing 100 mM phosphate buffer (NaH_2_PO_4_/Na_2_HPO_4_, to maintain the pH in the range of 4–8) at room temperature (24 ± 1 °C). The mixtures of secondary amines and carbonyl compounds were first prepared in phosphate buffer at defined concentrations and stabilized for at least 30 min to reach full equilibrium for the reactions of these two compounds. Then, nitrite was added to the solution to initiate the reaction. Sample aliquots were withdrawn at given time intervals, and nitrite was reduced to N_2_ by a common reducing agent, sulfamic acid, with a molar ratio of sulfamic acid:nitrite of 2:1.^25,28,38^ In the formation of *N*-nitrosamines, phosphate buffer in the concentration range of our study was reported to affect the reaction kinetics by less than a factor of 2.^39,40^ Therefore, the buffer effect was not further considered in the current study. All experiments were conducted at least in duplicates.

### Determination of Equilibrium Constants (*K*) for the Reactions of Secondary Amines with Formaldehyde

A titration method was used to fit the *K* values for the reactions of secondary amines with formaldehyde (Text S1 in the Supporting Information (SI)).^33,41,42^ Briefly, 10 μL aliquots of a formaldehyde solution (13.4 M) were added to 10 mL of a secondary amine solution (5 mM) with a micropipette, and pH changes were recorded. Based on this, the *K* value can be determined by plotting ΔpH as a function of [HCHO]_total_ (Figures S1–S5, Supporting Information). It should be noted that the determined *K* values are apparent equilibrium constants that take both formaldehyde and its hydrated form (methanediol) into account.

### Determination of Second-Order Rate Constants (*k*) for the Reactions of Nitrosating Agents with Secondary Amines, Carbinolamines, and Iminium Ions

Details for the determination of second-order rate constants are provided in the Supporting Information for reactions of iminium ions with NO_2_^–^ (Text S5), reactions of secondary amines with N_2_O_3_ (Text S6), and reactions of carbinolamines with N_2_O_3_ (Text S7).

### Kinetic Modeling

Kinetic modeling was conducted by Kintecus V6.70^43^ with the principle reactions and second-order rate constants obtained in this study and from the literature^44^ (Table S1, Supporting Information). This allowed us to evaluate the relative contribution of each pathway.

### Quantum Chemical Computations

Due to the instability of carbinolamines and iminium ions, direct validation of *N*-nitrosamine formation from these intermediates is currently infeasible. Thus, quantum chemical computations were conducted to identify embedded elementary reaction pathways and investigate if they are kinetically or thermodynamically feasible by calculating the aqueous-phase free energies of activation and reaction. Briefly, B3LYP/6-311+G(d,p) level of theory^45−47^ was used to calculate the wave function for a given molecular structure and calculate the frequencies in both the gas and aqueous phases. B3LYP/611+G(d,p) was successfully applied for nitrosating agents previously.^48^ The aqueous-phase structures and frequencies were obtained using an implicit polarizable continuum model (SMD).^49^ The validation of the DFT functional and basis set with the SMD is provided in Text S9 in the SI. To ensure the correct assignment to a local minimum and a transition state, the harmonic vibrational frequency was analyzed. All transition states were verified by intrinsic reaction coordinates (IRCs) to ensure the connection among reactants, transition states, and products.^50^ All quantum chemical computations were performed with Gaussian 16 revision C.01.^51^

## Results and Discussion

### *N*-Nitrosamine Formation from Selected Amines and Nitrite: Role of Formaldehyde

**Figure 1:**
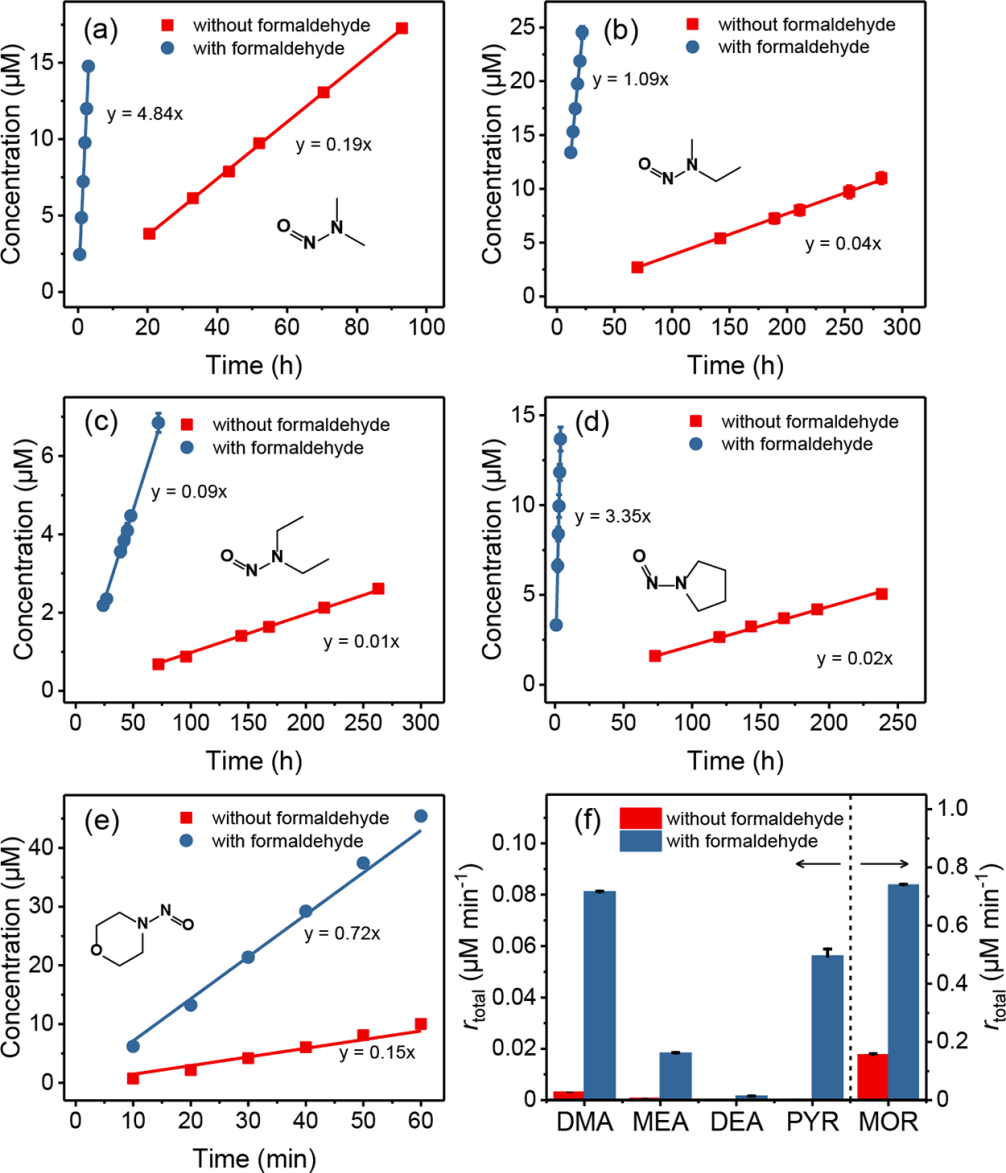
Nitrosation of selected secondary amines in the presence of nitrite. (a) *N*-Nitrosodimethylamine (NDMA), (b) *N*-nitrosomethylethylamine (NMEA), (c) *N*-nitrosodiethylamine (NDEA), (d) *N*-nitrosopyrrolidine (NPYR), and (e) *N*-nitrosomorpholine (NMOR) formation kinetics in the absence (red) and presence (blue) of formaldehyde. (f) Summary of the observed *N*-nitrosamine formation rates in the absence and presence of formaldehyde. The error bars are standard deviations from triplicate experiments. Experimental conditions: [secondary amine]_0_ = [formaldehyde]_0_ = 10 mM, [NO_2_^–^]_0_ = 50 mM, pH = 7, phosphate buffer = 0.1 M.

Figures 1a–e depict the *N*-nitrosamine formation kinetics resulting from the nitrosation of five secondary amines (10 mM) in the absence and presence of formaldehyde (10 mM) at pH 7 with a nitrite concentration of 50 mM. The observed rates of *N-*nitrosamine formation (*r*_total_) in the absence of formaldehyde are low for all secondary amines, except for MOR (Figure 1e). The formation kinetics decreased in the following order: NMOR (0.17 μM min^–1^) ≫ NDMA (3.09 × 10^–3^ μM min^–1^) > NMEA (6.41 × 10^–4^ μM min^–1^) > NPYR (3.69 × 10^–4^ μM min^–1^) > NDEA (1.62 × 10^–4^ μM min^–1^). In the presence of nitrite, nitrosation of secondary amine occurs mainly through N_2_O_3_ (1.5 × 10^–13^ M in equilibrium with NO_2_^–^, eqs S4–S6 in Text S2) by a nucleophilic attack.^34^ Second-order rate constants for the reactions of N_2_O_3_ with the selected secondary amines were determined in this study (Text S6 and Figures S11 and 12) to be in the range of 2.3 × 10^7^ to 2.1 × 10^8^ M^–1^ s^–1^ (Table S4). This is comparable to previously reported values within a factor of 1.3 to 3.8 based on the same method with *N*-nitrosamine formation measurements.^28^ The p*K*_a_ of MOR (8.56) is much lower than those for the other four secondary amines (10.54–11.12). Therefore, at pH 7, the concentration of the neutral MOR is 2–3 orders of magnitude higher than those for the other four secondary amines, which in turn accelerates the reaction between the secondary amine and reactive nitrosating agents. The presence of formaldehyde for the same experimental conditions significantly enhanced the formation kinetics by a factor of approximately 26, 29, 10, 152, and 5 for NDMA, NMEA, NDEA, NPYR, and NMOR, respectively (Figure 1f). To this end, the role of formaldehyde in enhancing the nitrosation of amines warrants further investigations, which are presented in the following sections.

### Reactions between Secondary Amines and Formaldehyde

To elucidate the role of formaldehyde in the enhancement of a nitrosation process, the reactions between secondary amines and formaldehyde were investigated. First, quantum chemical computations were conducted to elucidate the role of formaldehyde and methanediol in the reactions with secondary amines due to coexistence of these two species in aqueous solution.^42^ As shown in Figure S16, the computed free energy profiles for the reactions of methanediol with secondary amines are extremely high (e.g., 48.6 kcal mol^–1^ for DMA), while they are relatively low for formaldehyde (e.g., 8.7 kcal mol^–1^ for DMA) and can be considered as thermodynamically favorable. This suggests that the reactions of methanediol with secondary amines proceed via a two-step pathway (methanediol dehydrates to formaldehyde first, which then reacts with secondary amines). Then, equilibrium constants for the reactions of formaldehyde with the selected secondary amines were determined by titration of amines with a formaldehyde solution (Figures S1–S5) and are provided in Table 1. The results can be divided into two groups with different characteristics for *K*_1_ and *K*_2_ (Scheme 1c). For group I (MEA and DEA), only *K*_1_ can be determined because *K*_2_ was too low to be estimated, while for group II (DMA, PYR, MOR) both *K*_1_ and *K*_2_ can be obtained. *K*_1_ values were determined to be 195, 30, 757, 347, and 826 M^–1^, for MEA, DEA, DMA, PYR, and MOR, respectively. The *K*_1_ of DMA is much higher than for MEA and DEA, which can be attributed to the stronger steric hindrance for the addition of formaldehyde to the latter secondary amines.^33^*K*_2_ in group II for the equilibrium between protonated secondary amines and formaldehyde was between 1.0 and 1.2 M^–1^, which is more than 2 orders of magnitude lower than *K*_1_. Based on these equilibrium constants and predicted p*K*_a_ values (Table 1),^52^ the distribution of each species in equilibrium for the reactions of secondary amines with formaldehyde at pH 4–8 can be estimated. This is shown in Figure S7 and Table S2. The concentration of neutral carbinolamines is significantly higher than the neutral secondary amines (by a factor of 2 to 10), which may be important for *N*-nitrosamine formation by the carbinolamine pathway.

**Table 1:** Equilibrium Constants for the Reactions between Secondary Amines and Formaldehyde^a^^,^^b^

compound		p*K*_a_		this study		reference^33^	
		amine	carbinolamine^c^	*K*_1_ M^–1^	*K*_2_ M^–1^	*K*_1_ M^–1^	*K*_2_ M^–1^
Group I	Methylethylamine	10.54^c^	8.17	195			
	diethylamine	11.02^33^	8.42	30		40	
Group II	Dimethylamine	10.78^33^	7.93	757^d^	1.2	1150	1.7
	pyrrolidine	11.16^33^	8.52	347^d^	1.0	690	1.4
	morpholine	8.56^33^	6.03	826	1.1	800	0.9

a Equilibrium constants (*K*_1_ and *K*_2_) according to Scheme 1c.

b The *K*_2_ values for methylethylamine and diethylamine were too low to be determined.

c Predicted by Chemicalize software.^52^

d The *K*_1_ of dimethylamine and pyrrolidine is lower than previously reported values, deviation is in the range found among studies from different laboratories. The difference may be due to the different ionic strengths used in this study (25 mM), compared to that in the previous study (25–75 mM).^33^

### Kinetics and Mechanisms of the Enhanced *N*-Nitrosamine Formation

The pH significantly affects the speciation of reactive nitrosating species (N_2_O_3_/NO_2_^–^) and the equilibrium concentrations of secondary amines and intermediates (carbinolamines and iminium ions). For instance, the reactions of secondary amines and carbinolamines with N_2_O_3_ are unfavorable at high pH due to extremely low concentrations of N_2_O_3_ (1.5 × 10^–15^ M^–1^ at pH 8 compared to 1.5 × 10^–9^ M^–1^ at pH 4 for 50 mM nitrite, Table S3). These are ideal conditions for the elucidation of the iminium ion pathway. In contrast, at a low pH, the contributions of secondary amines and carbinolamines to *N*-nitrosamine formation will outcompete the iminium ion pathway. Therefore, the enhancement of *N*-nitrosamine formation kinetics and mechanisms can be assessed based on (1) the iminium ion pathway at high pH, (2) the carbinolamine pathway at low pH, (3) quantum chemical computations, and (4) kinetic simulations of NDMA formation as a function of pH.

#### Iminium Ion Pathway – N-Nitrosamine Formation from the Reaction of Iminium with Nitrite

**Figure 2:**
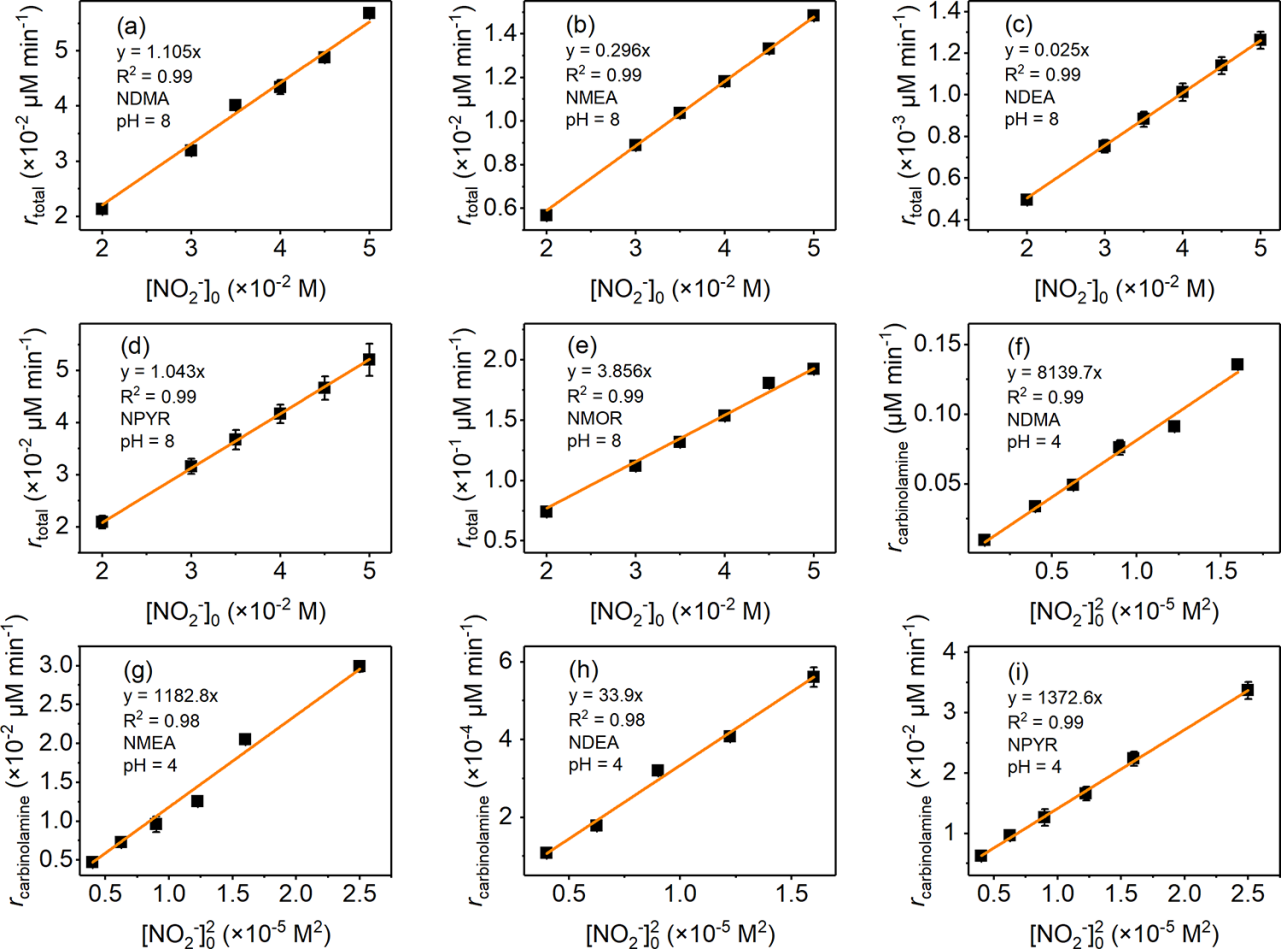
Nitrosation of (a–e) secondary amines (pH 8) by nitrite and (f–i) carbinolamines (pH 4) by N_2_O_3_ in the presence of formaldehyde. Linear relationship between (a–e) *r*_total_ and [NO_2_^–^]_0_ (iminium ion pathway), (f–i) *r*_carbinolamine_ and [NO_2_^–^]^2^_0_ (carbinolamine pathway). Experimental conditions: [secondary amine]_0_ = [formaldehyde]_0_ = 10 mM, [NO_2_^–^]_0_ = 1–50 mM, pH = 4 or 8, phosphate buffer = 0.1 M.

Figures 2a–e show a correlation between the observed *N*-nitrosamine formation kinetics (*r*_total_) and the nitrite concentration ([NO_2_^–^]) at pH 8 in the presence of formaldehyde. Generally, a linear increase of *r*_total_ as a function of the [NO_2_^–^] was observed. These results align well with a previously proposed mechanism that iminium ions are dominant intermediates under basic conditions.^30,37^ Iminium ions undergo rapid hydrolysis in aqueous solution,^32^ and a direct experimental determination of the second-order rate constants for the reactions of iminium ions with nitrite (*k*_iminium ion_) is currently not possible. However, iminium ions are in equilibrium with carbinolamines (*K*_3_ = [iminium]/[carbinolamine], eq S13 in Text S5). Therefore, *k*_iminium ion_ × *K*_3_ values (see detailed information in Text S5) were used as alternatives to *k*_iminium ion_ in kinetic evaluation and are provided in Table S4. Using this indirect approach, the contribution of the iminium ion pathway to *N*-nitrosamine formation can be estimated (Text S8), as illustrated in Figures S8 and S9. The iminium ion pathway is dominant under neutral and alkaline conditions. However, for more acidic conditions, the enhancement in the formation kinetics of *N*-nitrosamine cannot be fully explained by the iminium ion pathway (*r*_iminium ion_), except for MOR, as shown in Figures S8 and S9. This indicates that other pathways (carbinolamine pathway; see next section) may also be important in the nitrosation process.

#### Carbinolamine Pathway −N-Nitrosamine Formation from the Reaction of Carbinolamine with N_2_O_3_

Figures 2f−i show the correlation between the observed *N*-nitrosamine formation kinetics from the carbinolamine pathway (*r*_carbinolamine_) (by subtracting the *r*_iminium ion_ and *r*_R2NH_ from *r*_total_, eq S19 in Text S7) and the square of nitrite concentrations ([NO_2_^–^]^2^) in the range of 1 to 5 mM at pH 4 (see detailed information in Text S7). The linear relationship between *r*_carbinolamine_ and [NO_2_^–^]^2^, which is proportional to N_2_O_3_ (Text S2, eqs S4–S6), suggests that N_2_O_3_ may act as the nitrosating agent, similar to the secondary amine pathway (eq 4, Figures S11 and S12). The estimated second-order rate constants for the reactions of carbinolamines with N_2_O_3_, shown in Table S4, were on the same order of magnitude as those for secondary amines. Meanwhile, due to the much lower p*K*_a_ of carbinolamines, the estimated relative concentrations of neutral carbinolamines are generally higher than for neutral secondary amines (Table S2 and Figure S7), which may enhance the formation of *N*-nitrosamine under acidic conditions. Based on the fact that *N*-nitrosamine formation is enhanced in the presence of formaldehyde, carbinolamine seems to be a precursor at lower pH. This is supported by quantum chemical computations and kinetic simulations discussed in the next sections.

### Quantum Chemical Computations and Proposed *N*-Nitrosamine Formation Mechanism in the Presence of Formaldehyde

Quantum chemical computations were performed to reveal embedded pathways induced by secondary amines, iminium ions, and carbinolamines via two nitrosating agents (NO_2_^–^ and N_2_O_3_) for the formation of *N*-nitrosamines in the absence and presence of formaldehyde.

#### Secondary Amine Pathway

**Figure 3:**
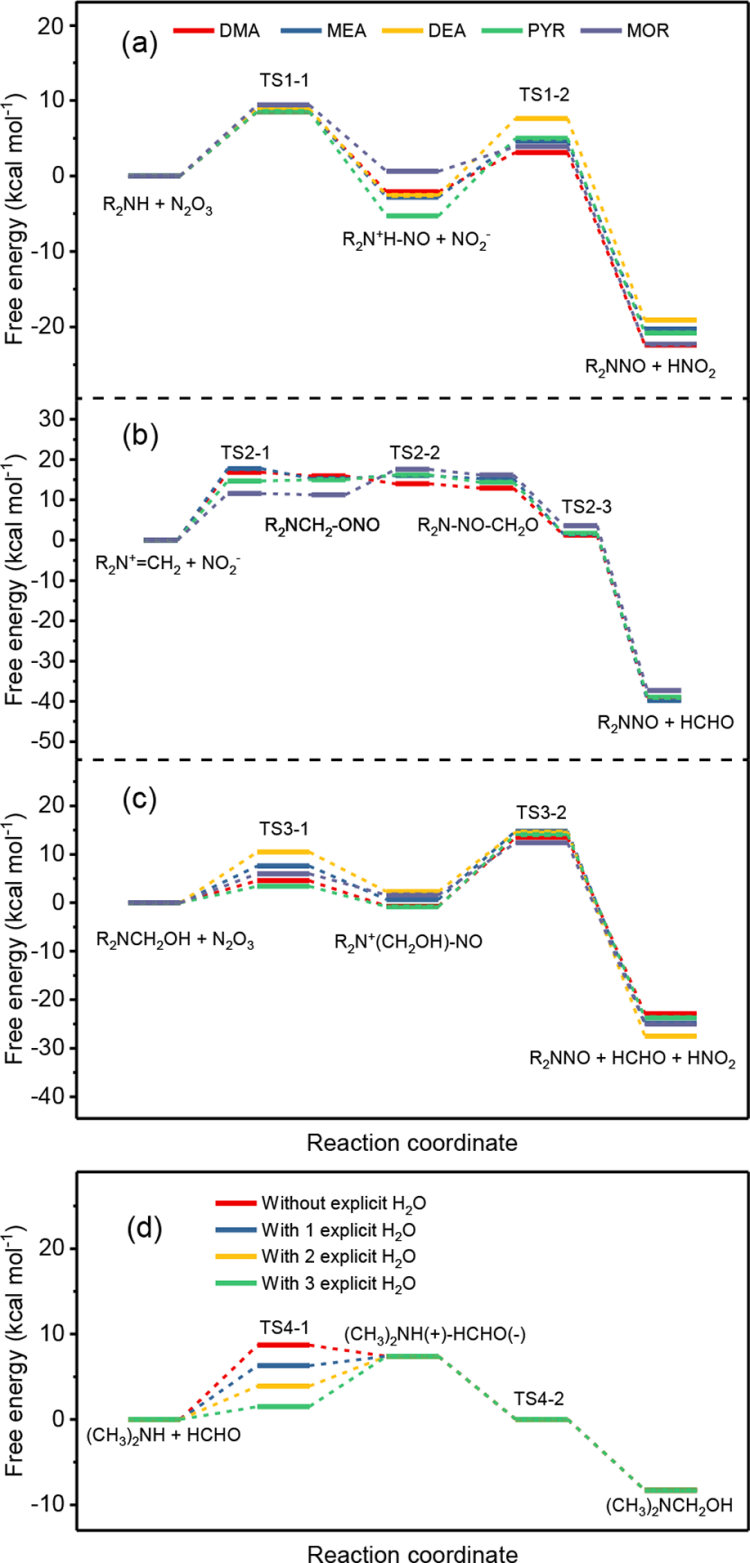
Calculated minimum energy pathway of nitrosation process from reactions of (a) secondary amine, (b) iminium ion, and (c) carbinolamine with the corresponding nitrosating agents and (d) carbinolamine formation from the reaction of dimethylamine (DMA) with formaldehyde (R represents alkyl groups attached to nitrogen).

As shown in Figure 3a, the aqueous-phase free energy profiles for the reactions of neutral secondary amines with N_2_O_3_ were computed. Although a previous study showed a one-step reaction for the formation of NDMA from the reaction of DMA with N_2_O_3_,^48^ our theoretical results suggest that this pathway proceeds via two steps: (1) electrophilic attack of the amine nitrogen by the NO group of N_2_O_3_ for the formation of intermediates, R_2_N^+^H-NO + NO_2_^–^, with free energies of activation between 8.5–9.4 kcal mol^–1^ and (2) a H-atom transfer reaction from the amine-nitrogen to the O-atom of NO_2_^–^ with free energies of activation between 3.3–10.3 kcal mol^–1^. When secondary amines are in their protonated forms, the free energies of activation of the first step are significantly higher in the range of 39.1–42.1 kcal mol^–1^ (Figure S13), making this species practically unreactive, in agreement with a previous report.^28^

#### Iminium Ion Pathway

The reactions of iminium ions with nitrite were found to proceed via a three-step pathway, as shown in Figure 3b: (1) electrophilic attack at the unsaturated carbon of iminium ions by the O-atom of NO_2_^–^ for the formation of an intermediate, R_2_NCH_2_–ONO, with free energies of activation between 11.6–17.8 kcal mol^–1^; (2) O-atom transfer from the ONO to the C-atom for the formation of R_2_N(NO)–CH_2_O with lower free energies of activation between −2.0–6.3 kcal mol^–1^; and (3) leaving of formaldehyde from the intermediate and formation of the corresponding *N*-nitrosamine with negative free energies of activation. Iminium ions can undergo fast hydrolysis to generate carbinolamines and are in equilibrium with them in water (Scheme 1c).^32^ Although this process is reversible and exergonic, significantly higher free energies of activation (e.g., up to 37.9 kcal mol^–1^ for DMA, Figure S14) resulted in a slow conversion rate from carbinolamine back to the corresponding iminium ion. This is in good agreement with the low concentrations of iminium ions in aqueous solution.^32^

#### Carbinolamine Pathway

The reactions between carbinolamines and N_2_O_3_ also proceed through a two-step process. The free energies of activation for the first step of an electrophilic attack at the amine-N by NO of N_2_O_3_ generating intermediates R_2_N^+^(CH_2_OH)–NO are in the range of 3.4–10.5 kcal mol^–1^ (Figure 3c), comparable to those of secondary amines. These results are also in accordance with the similar second-order rate constants determined for the reactions of secondary amines and carbinolamines with N_2_O_3_. Significantly larger free energies of activation for the reaction of carbinolamines with NO_2_^–^ (approximately 55 kcal mol^–1^, Figure S15) further support that NO_2_^–^ does not contribute to the enhanced formation of *N*-nitrosamines under acidic conditions. The intermediates undergo simultaneous cleavage of a C–H bond and structural arrangement to form *N*-nitrosamines, formaldehyde, and HNO_2_ (Figure 3c) with free energies of activation ranging from 10 to 13.5 kcal mol^–1^. For the formation of carbinolamines from the reaction of neutral secondary amines with formaldehyde, computed free energies of activation range from 8.7 to 11.2 kcal mol^–1^ (Figure S16), indicating little enhancement of carbinolamines formation. However, by addition of 1–3 explicit water molecules to account for explicit hydrogen bonds representing the hydration of formaldehyde, the free energies of activation for the reaction with DMA are reduced to 6.3 kcal mol^–1^ (one H_2_O molecule) and 1.5 kcal mol^–1^ (three H_2_O molecules) compared to 8.7 kcal mol^–1^ without H_2_O molecules, supporting the significant enhancement of NDMA formation by including explicit hydrogen bond(s) in a hydration process (Figure 3d).

### Kinetic Simulations of NDMA Formation

Based on the rate constants for the reactions of secondary amines and carbinolamines with N_2_O_3_ (at pH 4) and iminium ions with nitrite (at pH 8) obtained in this study (Table S4), a kinetic simulation of NDMA formation in the presence of formaldehyde was performed in a wider range of pH (from 4 to 8) and nitrite concentrations (from 1 to 50 mM) with the Kintecus software (Table S1).^43^ In addition, the reaction of DMA with formaldehyde for the production of carbinolamine was considered in the simulation procedure to obtain a better prediction.^44^Figure S17 shows that the predicted *r*_total_ fitted well with the experimental results at all of the nitrite concentrations and pH values tested. The carbinolamine pathway was found to dominate NDMA formation under acidic conditions, while the iminium ion pathway was the key contributor under neutral and alkaline conditions in kinetic simulation, which also agrees with the experimental observations in the previous sections.

At pH 4, we also observed a slower increase of *r*_carbinolamine_ as a function of [NO_2_^–^]^2^ at higher nitrite concentrations (5–50 mM), both in the simulated and experimental results (Figure S18a), which is contrary to the linear tendency at lower nitrite concentrations (<5 mM, Figure 2). To address this issue, the evolution of carbinolamine during nitrosation at pH 4–8 was simulated with high nitrite concentrations (50 mM) to elucidate the possible mechanism. As shown in Figure S19a, the simulated carbinolamine concentrations at pH 4 in the absence of nitrite are 4.9-fold higher than those in the presence of nitrite. The reaction of DMA with formaldehyde was reported to be relatively fast (*k* = 5.6 × 10^3^ M^–1^ s^–1^) and usually complete within seconds.^44^ Therefore, the lower simulated carbinolamine concentrations at the lower pH indicate a faster depletion by reaction with N_2_O_3_ under acidic conditions. This effect is enhanced by an increased N_2_O_3_ concentration (carbinolamine consumer) by a factor of ∼10^8^, from 1.5 × 10^–15^ to 1.2 × 10^–7^ M when the pH decreased from 8 to 4 (Table S3).

### *N*-Nitrosamine Formation in the Presence of Other Carbonyl Compounds

**Figure 4:**
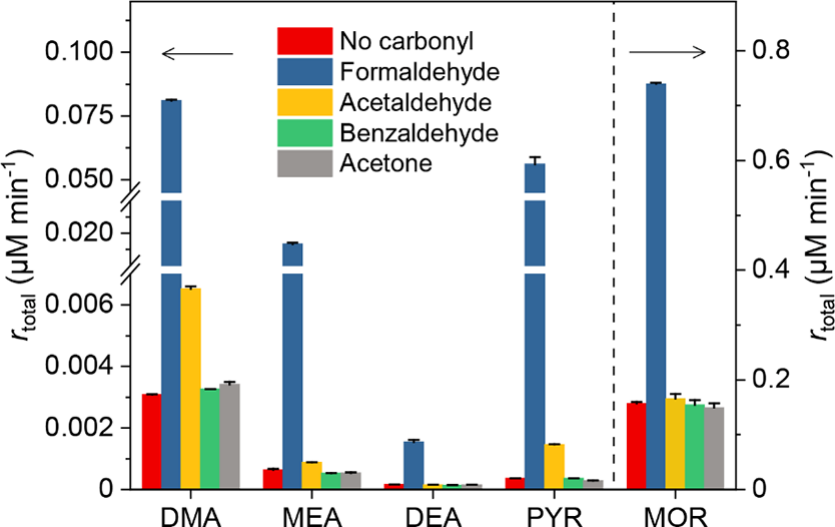
Effect of various carbonyl compounds on the rates of *N*-nitrosamine formation from nitrosation of secondary amines in ultrapurified water. Experimental conditions: [secondary amine]_0_ = [carbonyl compounds]_0_ = 10 mM, [NO_2_^–^]_0_ = 50 mM, pH = 7, and phosphate buffer = 0.1 M.

In addition to formaldehyde, other carbonyl compounds are also detected in engineered aquatic systems.^17,53−58^ For instance, formaldehyde, acetaldehyde, and acetone account for about 90% of the total carbonyl compounds detected in water reuse systems employing ozonation, with formaldehyde being the dominant species.^17^ Their concentrations range from <20 μg/L in drinking water^17^ to hundreds of mg/L in industrial effluent.^16^ As shown in Figure 4, compared to formaldehyde, only acetaldehyde showed a slight enhancement effect on *N*-nitrosamine formation. Benzaldehyde and acetone did not have an effect on the nitrosation reaction of the selected secondary amines, consistent with previous results.^30^ As discussed in the previous sections, iminium ions are the key intermediates that promote *N*-nitrosamine formation under neutral pH conditions. Two reasons may explain the different catalytic activities among the different carbonyl compounds. First, among the tested carbonyls, formaldehyde is the strongest electrophile with small steric hindrances, which may result in higher concentrations of carbinolamines and iminium ions. This, in turn, may enhance the *N*-nitrosamine formation. Second, the reactivity of iminium ions with nitrite is determined by their electrophilicities.^59^ An empirical structure–reactivity relationship (Text S5 and Table S5) suggests that the second-order rate constant for the reaction of the formaldehyde-derived iminium ion with nitrite is approximately 2 orders of magnitude higher than that for benzaldehyde. Although such a semiquantitative method is typically applied in organic solvents and has some uncertainties (1–2 orders of magnitude), this assessment can still partially explain the difference in the enhancement effects of different carbonyl compounds.^60^ For the reactions of DMA with other carbonyl compounds such as acetaldehyde, benzaldehyde, and acetone for the formation of NDMA, the computed free energies of activation are 16.7, 19.7, and 47 kcal mol^–1^, respectively (Figure S20). These activation energies are significantly higher compared with the reaction with formaldehyde (8.7 kcal mol^–1^), which is consistent with the experimental observations presented in Figure 4. Future efforts to further elucidate the effects of functional groups on carbonyl compounds (steric hindrance and electronic properties) on *N*-nitrosamine formation will help to better understand the underlying mechanisms.

### Practical Implications

The results from this study demonstrate the catalytic roles of carbonyl compounds in the conversion of amine precursors to *N*-nitrosamines via the iminium ion and carbinolamine pathways. Carbonyl compounds may be present in aquatic systems from incomplete removal in water treatment processes (at low levels),^34,61^ industrial discharges (at medium levels),^62^ and industrial production processes (e.g., Petasis reaction, using amines and carbonyl compounds at high levels as substrates for the production of amino acids).^63^ Kinetic modeling was performed to evaluate the specific potential for NDMA formation in these scenarios (Text S10). In typical water treatment processes (Scenario 1), the presence of trace amounts of formaldehyde (5 μM) may lead to a 10^4^-fold higher NDMA formation (Table S8), in which iminium ion acts as the key active intermediate for enhancement (Figure S21). However, the potential for NDMA formation (<0.1 ng/L) will still be far below any regulatory threshold (e.g., 100 ng/L in WHO guidelines) for NDMA due to insufficient precursor concentrations for nitrosation reaction.^10^ When the concentrations of these precursors increase to medium levels in industrial discharges (Scenario 2), the presence of formaldehyde results in NDMA formation potentials in the μg/L level range (Table S9) exceeding the WHO guidelines for all simulated conditions. In this scenario, the iminium ion is the key promoter at high pH and carbinolamine also participates in the NDMA formation at low pH (Figure S22). The worst scenario can be assumed for industrial production processes (Scenario 3). High DMA concentrations (0.1 M) may be used in synthetic processes, and traces of nitrite were modeled to produce negligible quantities of NDMA (<0.01 ng/L, Table S10). However, when formaldehyde is copresent as a reagent (Scenario 3-2 in Table S10), the potential for NDMA formation may exceed thousands of nanograms per liter, where the iminium ion is supposed to be the main driver for the enhanced nitrosation reaction (Figure S24). Meanwhile, when nitrite is used as a reagent (Scenario 3-3 in Table S10), trace quantities of DMA and formaldehyde may also lead to high concentrations of NDMA (>1.2 × 10^5^ ng/L), catalyzed by both the carbinolamine and iminium ion pathways (Figure S25). A recent survey on industrial discharges in Switzerland also detected larger concentrations of *N*-nitrosamines from chemical/pharmaceutical synthesis processes, which is in agreement with the modeled results in the current study.^22^ Overall, the highest risk of *N*-nitrosamine formation can be attributed to industrial wastewaters, and mitigation strategies should be implemented at this source.

Aldehydes/nitrite are common components in some engineered systems impacted by industrial activities. For instance, bronopol, an antifouling agent in industrial cooling or hydraulic fracturing processes, may degrade into formaldehyde and nitrite and contribute to *N*-nitrosamine formation when mixed with water containing secondary amines.^23,24^ In amine-based CO_2_ capture systems, flue gas containing high concentrations of nitrite and aldehydes can also pose a risk for unintended formation of *N*-nitrosamines.^25^ Considering the coexistence of numerous other amines and carbonyl compounds in aqueous solutions, the total *N*-nitrosamine formation might exceed the predicted levels reported in this study.

## Supporting Information Available

Determination of the equilibrium constants for secondary amines-formaldehyde reactions; speciation of reaction systems; estimation of p*K*_a_ values; determination of second-order rate constants for iminium ions, secondary amines, and carbinolamines; contribution of pathways to nitrosamine formation; validation of the theoretical method; estimation of NDMA formation under realistic conditions; titration of secondary amines with formaldehyde; speciation calculations for nitrite/nitrous acid and secondary amines/carbinolamine; estimation of the contributions of each pathway to nitrosamine formation; predicted *k*_obs_ for the iminium ion pathway; correlations between *k*_obs_ and the square of the nitrite concentration; quantum chemically computed calculated minimum energy pathways; comparison of experimental and modeling results for NDMA formation from various pathways; kinetic modeling of the evolution of carbinolamine; modeling of NDMA formation for realistic conditions; reactions used in the modeling; speciation of secondary amine/formaldehyde and nitrite/nitrous acid; summary of second-order rate constants; estimated p*K*_a_ values for MEA and carbinolamines; comparison of energies of activation, enthalpies of activation, and Gibbs free energies of activation; and summary of simulated NDMA formation for realistic conditions (PDF)
