# Supplementary material for: Role of Carbonyl Compounds for N-Nitrosamine Formation during Nitrosation: Kinetics and Mechanisms
Source: Environ Sci Technol. 2024 Mar 1;58(10):4792–801. doi: 10.1021/acs.est.3c07461 (PMC10938875; doi:10.1021/acs.est.3c07461)
Supplement: Supplementary file 1 — es3c07461_si_001.docx [file es3c07461_si_001.docx]

**Supplementary Information**

**Role of Carbonyl Compounds for *N*-nitrosamine Formation during Nitrosation: Kinetics and Mechanisms**

Yishuai Pan^1,2^, Florian Breider^1^, Benjamin Barrios^3^, Daisuke Minakata^3^, Huiping Deng^2^, Urs von Gunten^1,4*^

^1^ School of Architecture, Civil and Environmental Engineering (ENAC), Ecole Polytechnique Fédérale Lausanne (EPFL), CH-1015 Lausanne, Switzerland

^2^ Key Laboratory of Yangtze River Water Environment, Ministry of Education, Shanghai Institute of Pollution Control and Ecological Security, College of Environmental Science and Engineering, Tongji University, Shanghai, 20092, China

^3^ Department of Civil, Environmental and Geospatial Engineering, Michigan Technological University, 1400 Townsend Drive, Houghton, Michigan 49931, USA.

^4^ Eawag, Swiss Federal Institute of Aquatic Science and Technology, CH-8600 Dübendorf, Switzerland

Corresponding author: Urs von Gunten (vongunten@eawag.ch)

**This file includes 10 texts, 25 figures, 10 Tables and 52 pages.**

**Text S1.** Determination the equilibrium constants (*K*) for the reactions of secondary amines with formaldehyde.

*K*_1_ and *K*_2_ in eq. S1 (Scheme 1c in the main text) for the reaction of secondary amines with formaldehyde in aqueous solution were determined by monitoring the pH changes as a function of formaldehyde concentration by a titration method^1^, i.e., the relative pH changes (△pH) during the addition of formaldehyde to the secondary amine solutions. Specifically, 5 mM secondary amine solutions were prepared in 20 mL amber borosilicate bottles, and the pH values were adjusted to their p*K*_a_ values (pH_0_, 50% free base), then 13.4 M formaldehyde was added to the secondary amine solutions dropwise (10 μL/drop) and the pH (pH_t_) for each addition was recorded. Finally, the relationship between △pH (△pH = pH_t_ - pH_0_) and [HCHO]_total_ was fitted by eq. S2 (for MEA and DEA) and eq. S3 (for DMA, PYR and MOR) in Figures S1 - S5. The reason for using different equations is that *K*_2_ values for MEA and DEA were too small to be estimated by the titration procedure, thus were not considered.


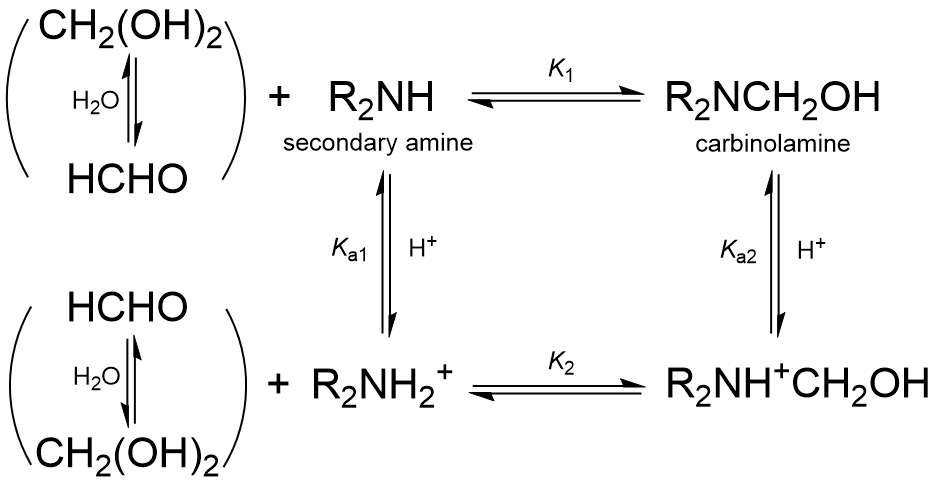
 (S1)

| △pH = log (1 + *K*_1_[HCHO]_total_) | (S2) |
| --- | --- |
| △pH = log (1 + *K*_1_[HCHO]_total_) - log (1 + *K*_2_[HCHO]_total_) | (S3) |

Eqs. S2 or S3 were used to fit *K* values for the reactions of different secondary amines with formaldehyde because of the following reasons: (1) During the titration procedure, several reactions may occur, as shown in eq. S1 and also in previous publications^1^: (i) formaldehyde is in equilibrium with methanediol, while methanediol dehydrates to formaldehyde first and then reacts with the neutral secondary amine (*K*_1_); (ii) protonation of secondary amine (*K*_a1_); (iii) protonation of carbinolamine (*K*_a2_). If we already know the value of *K*_1_, *K*_a1_ and *K*_a2_, then *K*_2_ will be fixed as these values are not independent (i.e., *K*_1_/*K*_2_ = *K*_a2_/*K*_a1_). In this case, if a protonation of carbinolamine occurs, then the reaction of the protonated secondary amine with formaldehyde is also possible. Typically, *K*_2_ is 2-3 orders of magnitude lower than *K*_1_, confirmed by a previous publication.^1^ This means, even though the reaction of the protonated secondary amine with formaldehyde might occur, the equilibrium constant is extremely low. Therefore, eq. S3 is the first choice for the fitting procedure, as it includes the protonation of carbinolamine while eq. S2 does not. (2) However, if we use eq. S3, very poor fitting results are obtained for MEA and DEA, which indicates that *K*_2_ for these two secondary amines are too small to be accurately fitted. When we used eq. S2 (protonation of carbinolamine was not considered) to fit the experimental results, excellent fitting results were obtained in accordance with a previous publication.^1^ (3) We also plotted antilog △pH against [HCHO]_total_ for these reactions, which clearly shows that eq. S2 is more suitable for DEA and MEA, while eq. S3 is more suitable for DMA, PYR and MOR.

**Text S2.** Speciation of HNO_2_, NO_2_^-^ and N_2_O_3_ in aqueous solution.

The speciation of a system containing nitrite is given by eqs. S4-S6 including the acid-base equilibrium (eq. S4, p*K*_a_) and equilibrium between HNO_2_ and N_2_O_3_ (eq. S5, *K*_N2O3_). The speciation results are provided in Figure S6 and Table S3.

| HNO_2_ ⇄ NO_2_^-^ + H^+^ | p*K*_a_ = 3.15 (S4) |
| --- | --- |
| 2HNO_2_ ⇄ N_2_O_3_ + H_2_O | *K*_N2O3_ = 3 × 10^-3^ M^-1^ (S5) |
| [N_2_O_3_] = *K*_N2O3_([NO_2_^-^][H^+^]/*K*_a_)^2^ | (S6) |

**Text S3.** Estimation of the p*K*_a_ for MEA and carbinolamines

The p*K*_a_ values for MEA and carbinolamines were estimated using the Chemicalize software (ChemAxon, <https://chemaxon.com/products/chemicalize>) and the results are provided in Table S6.

**Text S4.** Speciation of solutions containing secondary amines and formaldehyde.

In solutions containing secondary amines and formaldehyde, there are dissociation equilibria for secondry amines (eq. S7), reactions between secondary amines and formaldehyde (eq. S8) and protonation of carbinolamines (eq. S9). A mass balance for secondary amines-containing species (eq. S10) and formaldehyde-containing species (eq. S11) can also be established. In these equations, the p*K*_a_ for MEA in eq. S7 and carbinolamines in eq. S9 are predicted by the Chemicalize software (Text S3), *K* values for eq. S8 were obtained by a titration method (Text S1). By programming these equations in Matlab 2017a and entering the initial concentrations of secondary amines and formaldehyde, the equilibrium concentrations of each species can be obtained by solving eqs. S7 – S11. The corresponding results are provided in Table S2 and Figure S7.

| R_2_NH_2_^+^ ⇄ R_2_NH + H^+^ | (S7) |
| --- | --- |
| R_2_NH + HCHO_total_ ⇄ R_2_NCH_2_OH | (S8) |
| R_2_NH^+^CH_2_OH ⇄ R_2_NCH_2_OH + H^+^ | (S9) |
| [R_2_NH_2_^+^]_0_ + [R_2_NH]_0_ = [R_2_NH_2_^+^] + [R_2_NH] + [R_2_NCH_2_OH] + [R_2_NH^+^CH_2_OH] | (S10) |
| [HCHO]_total,0_ = [HCHO]_total_ + [R_2_NCH_2_OH] + [R_2_NH^+^CH_2_OH] | (S11) |

[R_2_NH_2_^+^]_0_, [R_2_NH]_0_ and [HCHO]_total,0_ are initial concentrations of protonated secondary amines, deprotonated secondary amines and the sum of formaldehyde and its hydrated form (methanediol), respectively. [R_2_NH_2_^+^], [R_2_NH], [R_2_NH^+^CH_2_OH], [R_2_NCH_2_OH], and [HCHO]_total_ are equilibrium concentrations of protonated secondary amines, deprotonated secondary amines, protonated carbinolamines, deprotonated carbinolamines and the sum of formaldehyde and its hydrated form (methanediol), respectively.

**Text S5.** Determination and estimation of second-order rate constants (*k*_iminium ion_) for the reactions of iminium ions with nitrite.

This text can be separated in the following 2 sections:

(1) Section 1: Using the value (*k*_iminium ion_×*K*_3_) as an alternative to *k*_iminium ion_.

From a mechanism proposed by Keeper and Roller in 1973, the formation of *N*-nitrosamine is mainly attributed to the reaction between iminium ions and nitrite under basic conditions.^2, 3^. We also observed a linear increase of the formation rate of *N*-nitrosamine as a function of the nitrite concentration in the presence of formaldehyde at pH 8 (Figures 2a – e in the main text), agreeing with the previously proposed mechanism that iminium ions are the dominant intermediates under alkaline conditions. This indicates that the formation rate of *N*-nitrosamine through the iminium ion pathway (*r*_iminium ion_) at pH 8 can be expressed by eq. S12.

In eq. S12, *r*_iminium ion_ can be experimentally determined and [NO_2_^-^] is the concentration of nitrite. Iminium ion hydrolyzes rapidly and is in equilibrium with the corresponding carbinolamine, as shown in eq. S13. The equilibrium constant (*K*_3_) for eq. S13 cannot be experimentally determined, as the transient concentration of iminium ion is extremely low,^4^ resulting in the unknown concentration of iminium ion ([R_2_N^+^=CH_2_]) in eq. S12. This in turn means *k*_iminium ion_ in eq. S12 cannot be determined experimentally.

By substituting [R_2_N^+^=CH_2_] in eq. S12 with *K*_3_×[R_2_NH^+^CH_2_OH] in eq. S13, eq. S12 can be transformed to eq. S14. The concentration of protonated carbinolamine ([R_2_NH^+^CH_2_OH]) in eq. S14 can be determined experimentally (Text S4). *k*_iminium ion_×*K*_3_ in eq. S14 can be determined by plotting *r*_iminium ion_ as a function of the nitrite concentrations at pH 8, as shown in Figures 2a - e (data provided in Table S4). The slope was divided by [R_2_NH^+^CH_2_OH] to obtain (*k*_iminium ion_×*K*_3_).

In this study, (*k*_iminium ion_×*K*_3_) in eq. S14 was used as an alternative to the *k*_iminium ion_ in eq. S12 throughout the discussion, except for the section “*N*-nitrosamine Formation in the Presence of Other Carbonyl Compounds” in the main text. This enabled a kinetic simulation of *N*-nitrosamine formation from the iminium ion pathway.

| R_2_N^+^=CH_2_ + NO_2_^-^ 🡪 R_2_NNO + HCHO  *r*_iminium ion_ = *k*_iminium ion_[R_2_N^+^=CH_2_][NO_2_^-^] | (S12) |
| --- | --- |
| R_2_NH^+^CH_2_OH ⇄ R_2_N^+^=CH_2_ + H_2_O  *K*_3_ = [R_2_N^+^=CH_2_]/[R_2_NH^+^CH_2_OH] | (S13) |
| *r*_iminium ion_ = (*k*_iminium ion_×*K*_3_)[R_2_NH^+^CH_2_OH][NO_2_^-^] | (S14) |

Eq. S12 is the reaction between iminium ions and nitrite, eq. S13 is the equilibrium between iminium ions and carbinolamine, eq. S14 was derived by substituting [R_2_N^+^=CH_2_] in eq. S12 with *K*_3_[R_2_NH^+^CH_2_OH] in eq. S13.

(2) Section 2: estimation of *k*_iminium ion_ through an empirical relationship.

In the section “*N*-nitrosamine Formation in the Presence of Other Carbonyl Compounds” in main text, we further assessed the *N*-nitrosamine formation with other commonly detected carbonyl compounds. Only acetaldehyde showed a slight enhancement effect while others failed to promote the *N*-nitrosamine formation. Thus, determination of the *k*_iminium ion_ using the method described in Section 1 of Text S5 was impossible, as no kinetic data for the iminium ion pathway (*r*_iminium ion_) can be experimentally obtained.

Therefore, an empirical relationship was applied to correlate the logarithms of the second-order rate constants (log *k*) for reactions of iminium ions and nitrite at 20 ℃ by eq S15.^5^

log *k* = s_N_ (*E* + *N*) (S15)

*E* is the electrophilicity of electrophiles (iminium ions), *N* is the nucleophilicity of nucleophiles (nitrite) and s_N_ refers to a “nucleophile-specific” parameter. Specific parameters for some *E*, *N*, and s_N_ are accessible on Mayr's Database of Reactivity Parameters (https://www.cup.lmu.de/oc/mayr/reaktionsdatenbank/).

Then, the *k*_iminium ion_ for the reactions of iminium ions with nitrite disscussed in this section can be estimated based on eq. S15 and they are summarized in Table S5.

It should be noted that this equation is semi-quantitative with an accuracy of 1-2 orders of magnitude. Eq. S15 is usually applied in organic solvents but can also be used for estimations and comparisons in aqueous solution^6^.

**Text S6.** Determination of second-order rate constants (*k*_R2NH_) for the reactions of secondary amines with N_2_O_3_.

*k*_R2NH_ was determined by eq. S17 by monitoring the *N*-nitrosamine formation rate (*r*_R2NH_) of specific secondary amines in the absence of catalysts with increasing nitrite concentrations. A set of experiments was conducted with increasing nitrite concentrations and *r*_R2NH_ was recorded. Then, *k*_R2NH_ can be determined from the slope of a plot of *r*_R2NH_ as a function of the square of the nitrite concentrations as shown in Figures S11-S12 (data is provided in Table S4). The slope was divided by *K*_N2O3_, [R_2_NH] and [H^+^] and multiplied by *K*_a_ to obtain *k*_R2NH_.

Eq. S16 is the reaction between secondary amines and N_2_O_3_, and eq. S17 is derived by substituting [N_2_O_3_] with *K*_N2O3_([NO_2_^-^][H^+^]/*K*_a_)^2^ from eq. S3. Note that the concentration of R_2_NH used in this section is 10 mM and the molar yield of *N*-nitrosamine formation is less than 1%, indicating that the concentration of R_2_NH throughout the nitrosation process can be assumed to be constant.

| R_2_NH + N_2_O_3_ ⇄ R_2_NNO + other product  *r*_R2NH_ = *k*_R2NH_[R_2_NH][N_2_O_3_] | (S16) |
| --- | --- |
| *r*_R2NH_ = *k*_R2NH_[R_2_NH]*K*_N2O3_([NO_2_^-^][H^+^]/*K*_a_)^2^ | (S17) |

**Text S7.** Determination of second-order rate constants (*k*_carbinolamine_) for the reactions of carbinolamines with N_2_O_3_.

In this study, the reaction solution contains a mixture of secondary amines, nitrite and aldehydes. The resulting products (carbinolamine and iminium ion) are always in equilibrium with secondary amines (Scheme 1c in the main text). There are no methods to separate them and determine *k*_carbinolamine_ for the reactions of N_2_O_3_ with carbinolamine directly. Thus, *k*_carbinolamine_ was determined from an indirect method as follows:

1. In the current study, the total observed formation rate of *N*-nitrosamine (*r*_total_) consists of three pathways (secondary amine (*r*_R2NH_), carbinolamine (*r*_carbinolamine_) and iminium ion (*r*_iminium ion_)) (eq. S18).
2. Thus, the observed formation rate of carbinolamines (*r*_carbinolamine_) can be obtained by subtracting *r*_R2NH_ and *r*_iminium ion_ from *r*_total_ (eq. S19). *r*_iminium ion_ and *r*_R2NH_ can be experimentally determined as outlined in Texts S5 and S6, respectively.
3. As evidenced in the main text (in the section “carbinolamine pathway”), the formation of *N*-nitrosamine from the carbinolamine pathway through the reaction with N_2_O_3_ is similar to the secondary amine pathway. Thus, *r*_carbinolamine_ can be expressed in a form similar to secondary amines (eq. S20), with a second-order rate constant for the reaction of carbinolamine with N_2_O_3_ (*k*_carbinolamine_) on the left side and concentrations of carbinolamine ([carbinolamine]) and N_2_O_3_ ([N_2_O_3_]) on the right side (eq. S20).
4. By substituting [N_2_O_3_] with *K*_N2O3_([NO_2_^-^][H^+^]/*K*_a_)^2^ from eq. S3, *r*_carbinolamine_ can be expressed as eq. S21.
5. Finally, *k*_carbinolamine_ can be determined by plotting *r*_carbinolamine_ against the square of nitrite concentrations as shown in Figures 2f - i (in the main text) (data shown in Table S4). The slope was divided by *K*_N2O3_, [carbinolamine] and [H^+^] and multiplied by *K*_a_ to obtain *k*_carbinolamine_.

It should be noted that the second-order rate constants (*k*_carbinolamine_) reported here are estimations based on the assumption that only secondary amine, carbinolamine and iminium ion contribute to the *N*-nitrosamine formation. The *k*_carbinolamine_ determined here has some uncertainties, because it is based on two other rate constants and the calculated carbinolamine concentration.

| *r*_total_ = *r*_R2NH_ + *r*_iminium ion_ + *r*_carbinolamine_  *r*_carbinolamine_ = *r*_total_- *r*_R2NH_ - *r*_iminium ion_ | (S18)  (S19) | |
| --- | --- | --- |
| Carbinolamine + N_2_O_3_ ⇄ R_2_NNO + other product  *r*_carbinolamine_ = *k*_carbinolaime_[carbinolamine] [N_2_O_3_] | (S20) | |
| *r*_carbinolamine_ = *k*_carbinolaime_[carbinolamine] *K*_N2O3_([NO_2_^-^][H^+^]/*K*_a_)^2^ | | (S21) |

*r*_total_, *r*_R2NH_, *r*_iminium ion_, and *r*_carbinolamine_ are pseudo first-order formation rate constants of the total pathway, for secondary amines, iminium ions, and carbinolamines, respectively. Eq. S20 corresponds to the reaction between carbinolamines and N_2_O_3_, and eq. S21 is derived by substituting [N_2_O_3_] in eq. S20 with *K*_N2O3_([NO_2_^-^][H^+^]/*K*_a_)^2^ from eq. S3.

**Text S8.** Contribution of each pathway (secondary amine, carbinolamine and iminium ion) to the total *N*-nitrosamine formation rate.

Based on the acquired second-order rate constants and known concentrations of each species, the contribution of each pathway to the observed formation rate can be expressed by eq. S22.

*r*_total_ = *r*_R2NH_ + *r*_iminium ion_ + *r*_carbinolamine_

= *k*_R2NH_[R_2_NH][N_2_O_3_] + (*k*_iminium ion_×*K*_3_) [R_2_NH^+^CH_2_OH][NO_2_^-^] + *k*_carbinolaime_[carbinolamine][N_2_O_3_] (S22)

It should be noted that the contributions of each pathway reported here are estimations based on the assumption that only secondary amine, carbinolamine and iminium ion contribute to the *N*-nitrosamine formation. The second-order rate constants for the secondary amine pathway (*k*_R2NH_) were experimentally determined directly, whereas, second-order rate constants for iminium ion ((*k*_iminium ion_×*K*_3_) as an alternative to *k*_iminium ion_) and carbinolamine (*k*_carbinolamine_) pathways are determined from indirect methods, as shown in Texts S5 and S7, respectively.

**Text S9.** Validation of the theoretical method

To validate the B3LYP^7-9^ exchange-correlation functional, the activation energies, enthalpies of activation, and free energies of activation for selected amines and N_2_O_3_ were calculated and benchmarked with those obtained by high level DFT or ab initio methods. Eqs. S23-S25 were employed to calculate the aqueous-phase energies of activation ($\Delta E_{\text{aq,calc}}^{\text{act}}$), enthalpies of activation ($\Delta H_{\text{aq,calc}}^{\text{act}}$), and Gibbs free energies of activation ($\Delta G_{\text{aq,calc}}^{\text{act}}$), respectively:

$\Delta E_{\text{aq,calc}}^{\text{act}}=E_{\text{0,aq}}(\text{TS})-[E_{\text{0,aq}}(\text{R}_{1}\text{R}_{2}\text{NH})+E_{\text{0,aq}}(\text{N}_{2}\text{O}_{3})]$ (S23)

$\Delta H_{\text{aq,calc}}^{\text{act}}=[E_{\text{0,aq}}(\text{TS})+H_{\text{corr,aq}}(\text{TS})]-[E_{\text{0,aq}}(\text{R}_{1}\text{R}_{2}\text{NH})+H_{\text{corr,aq}}(\text{R}_{1}\text{R}_{2}\text{NH})+E_{\text{0,aq}}(\text{N}_{2}\text{O}_{3})+H_{\text{corr,aq}}(\text{N}_{2}\text{O}_{3})]$ (S24)$\Delta G_{\text{aq,calc}}^{\text{act}}=[E_{\text{0,aq}}(\text{TS})+G_{\text{corr,aq}}(\text{TS})]-[E_{\text{0,aq}}(\text{R}_{1}\text{R}_{2}\text{NH})+G_{\text{corr,aq}}(\text{R}_{1}\text{R}_{2}\text{NH})+E_{\text{0,aq}}(\text{N}_{2}\text{O}_{3})+G_{\text{corr,aq}}(\text{N}_{2}\text{O}_{3})]$ (S25)

where $E_{\text{0,aq}}$,$H_{\text{corr,aq}}$, and $G_{\text{aq,calc}}^{\text{act}}$correspond to the aqueous-phase electronic energy, thermal correction of the enthalpy, and thermal correction of the Gibbs free energy of the species in parenthesis, respectively, and TS corresponds to transition state, and R_1_R_2_NH to the secondary amine. $H_{\text{corr,aq}}$and $G_{\text{aq,calc}}^{\text{act}}$ were obtained at the B3LYP/6-311+G(d,p) level of theory with the SMD solvation model^10^, and $E_{\text{0,aq}}$was calculated at different levels of theory over the B3LYP/6-311+G(d,p) geometries. The M06-2X exchange-correlation functional^11^ and the coupled cluster with single and double excitations with perturbative treatment of triple excitations, CCSD(T)^12^ were selected. The M06-2X functional has been shown to describe accurately the thermochemistry and kinetics of reactions involving organic compounds^11, 13^, and the CCSD(T) method is considered the “gold standard” for systems in the gas phase without multireference character.^14^ These theoretical results were compared and validated with experimental values reported in the literature.

Table S7 summarizes the values of $\Delta E_{\text{aq,calc}}^{\text{act}}$, $\Delta H_{\text{aq,calc}}^{\text{act}}$, and $\Delta G_{\text{aq,calc}}^{\text{act}}$for the reactions of dimethylamine, diethylamine, and morpholine with N_2_O_3_. At all levels of theory, the energies of activation were negative, indicating favorable formation of the transition states. For the three amines, the values of Δ*E*^act^_calc,aq_ at the M06-2X level of theory were on average 0.4 kcal/mol lower than at the B3LYP level of theory, and 2.9 kcal/mol lower at the CCSD(T) level of theory than at the B3LYP level of theory. These results agree with the tendency of B3LYP functional to overestimate energies relative to more expensive levels of theory.^11^

The results for Δ*H*^act^_calc,aq_ displayed the same trend as for Δ*E*^act^_calc,aq_ for the three amines. However, the values obtained with the B3LYP functional were slightly positive compared to the negative values obtained by the M06-2X functional or CCSD(T) level of theory. Casado et al^15^ reported experimental enthalpies of activation for dimethylamine, diethylamine, and morpholine, and while our calculated values with the B3LYP functional are on average 2.3 kcal/mol within the experimental values, the values obtained at the CCSD(T) level of theory are 4.9 kcal/mol. Furthermore, the CCSD(T) level of theory predicts negative enthalpies of activation, while the experimental values are slightly positive. We attribute this discrepancy to the loose character of the transition states, characterized by the small imaginary vibrational frequencies of -55.2 kcal/mol. Prior studies have provided experimental evidence that the reaction of secondary amines with N_2_O_3_ is diffusion-limited characterized by having activation enthalpies of 2.4-4.9 kcal/mol.^15, 16^ This information corroborates our difficulty in obtaining a transition state for the reaction of amines with N_2_O_3_, and with the small or negative values of Δ*H*^act^_calc,aq_ obtained at all levels of theory.

The values of Δ*G*^act^_calc,aq_ are more appropriate to study the reactivity as they capture steric factors and entropy changes in the formation of the transition states. The results for Δ*G*^act^_calc,aq_ for the three amines at different levels of theory follow the same trend: the M06-2X functional and the CCSD(T) level of theory are smaller than the values obtained with the B3LYP functional. Most importantly, the results obtained with the CCSD(T) level of theory reveal that there is no difference in reactivity between the three amines because their Δ*G*^act^_calc,aq_ values differ by only 0.7 kcal/mol. With the B3LYP functional, the values of Δ*G*^act^_calc,aq_ vary only 0.9 kcal/mol. These results are consistent with the idea that these reactions are diffusion-limited and that the rate does not vary between amines.^15, 16^ We conclude that the B3LYP functional accurately captures the trends in reactivity common to the five selected amines in this work.

**Text S10.** Estimation of NDMA formation under realistic conditions.

In the manuscript (section “Kinetic Simulation of NDMA Formation”), we apply kinetic modeling with equations from Table S1 to estimate the NDMA and carbinolamine formation as a function of the pH. Here, we used the same modeling procedure to model the NDMA formation under more realistic conditions to show the impacts of carbonyl compounds on NDMA formation.

**Scenario 1**: NDMA formation in typical water treatment process. In this scenario, DMA, formaldehyde and nitrite are present at low levels.

**Scenario 2**: NDMA formation in industrial discharges. In this scenario, DMA, formaldehyde and nitrite are present at intermediate levels.

**Scenario 3**: NDMA formation in an industrial production process. In this scenario, DMA, formaldehyde and nitrite can be present at different levels, from low to high concentrations. Specifically, they can be present as follows: (1) Trace nitrite with trace quantities of DMA and formaldehyde to model the situation when these reactants are present as impurities/intermediates during syntheses procedures; (2) trace nitrite with high concentrations of DMA and formaldehyde to model a situation when DMA and formaldehyde are used as reagents in a synthesis process but the water is polluted with traces of nitrite; (3) high nitrite concentration with trace concentrations of DMA and formaldehyde to model the presence of DMA and formaldehyde as impurities/intermediates during aqueous nitrosation reactions for chemical synthesis.

Detailed information of precursor concentrations, reaction conditions and NDMA formation potentials are provided in Tables S8 - S10 and Figures S21 - S25.


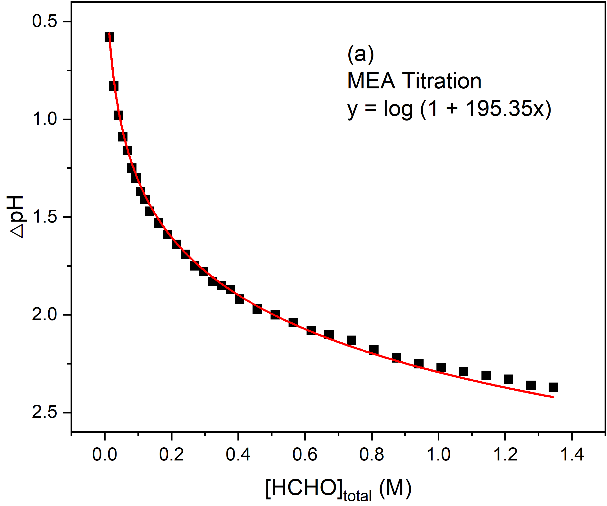

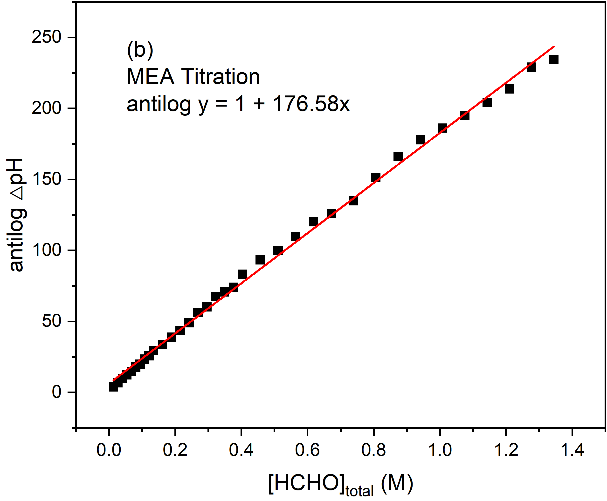


**Figure S1**. Titration of MEA with a formaldehyde solution. Measured change of (a) pH and (b) antilog pH as a function of the formaldehyde concentrations at 24 ± 1 ℃. The symbols represent the experimental data and the lines represent fits of the results based on eq. S2. Experimental conditions: [MEA]_0_ = 5 mM, 50% free base, [ionic strength] = 25 mM, [pH]_0_ = 10.54.


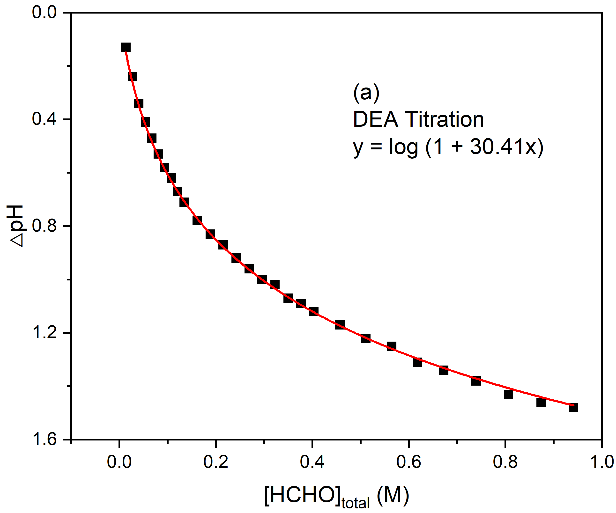

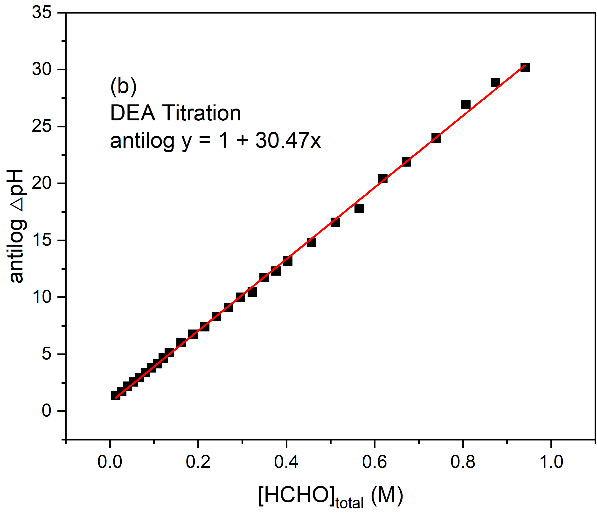


**Figure S2**. Titration of DEA with a formaldehyde solution. Measured change of (a) pH and (b) antilog pH as a function of the formaldehyde concentrations at 24 ± 1 ℃. The symbols represent the experimental data and the lines represent fits of the results based on eq. S2. Experimental conditions: [DEA]_0_ = 5 mM, 50% free base, [ionic strength] = 25 mM, [pH]_0_ = 11.02.

**
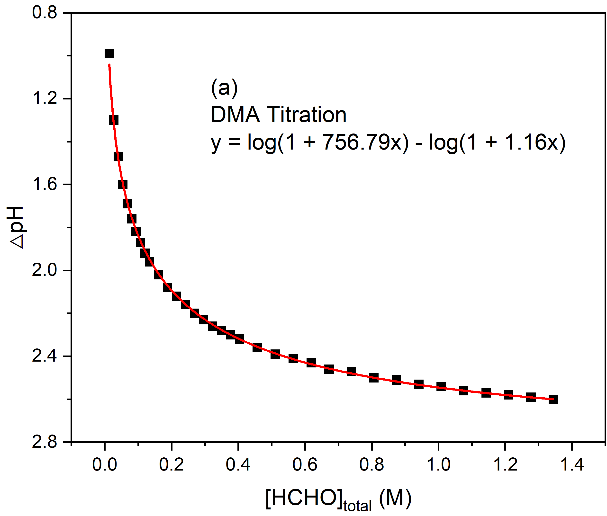

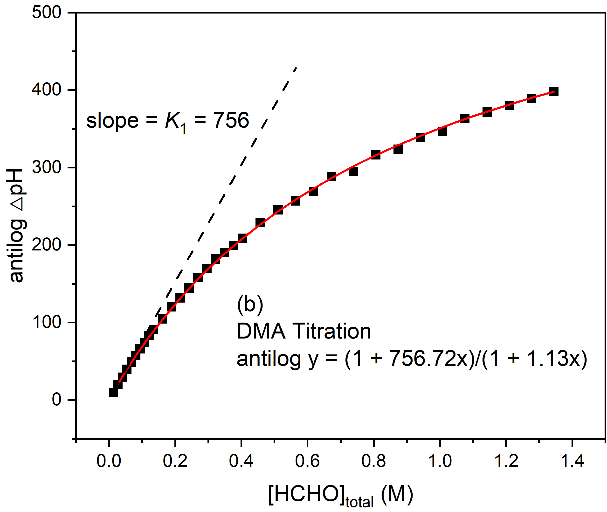
**

**Figure S3**. Titration of DMA with a formaldehyde solution. Measured change of (a) pH and (b) the antilog pH as a function of the formaldehyde concentrations at 24 ± 1 ℃. The symbols represent the experimental data and the lines represent fits of the results based on eq. S3. The dashed line in (b) is the predicted line based on the assumption without consideration of protonation of carbinolamine. Experimental conditions: [DMA]_0_ = 5 mM, 50% free base, [ionic strength] = 25 mM, [pH]_0_ = 10.87.


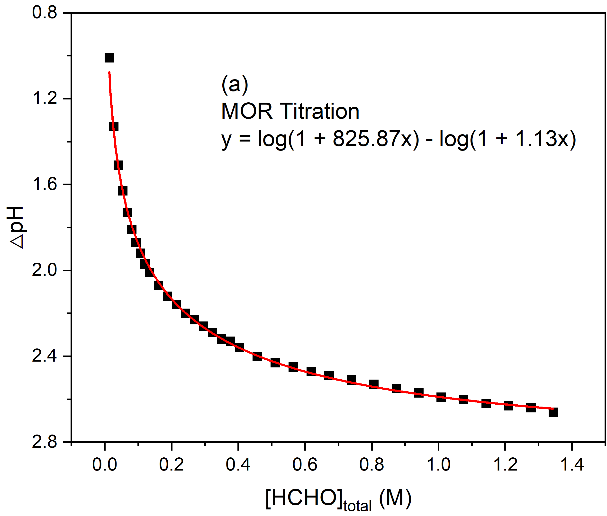

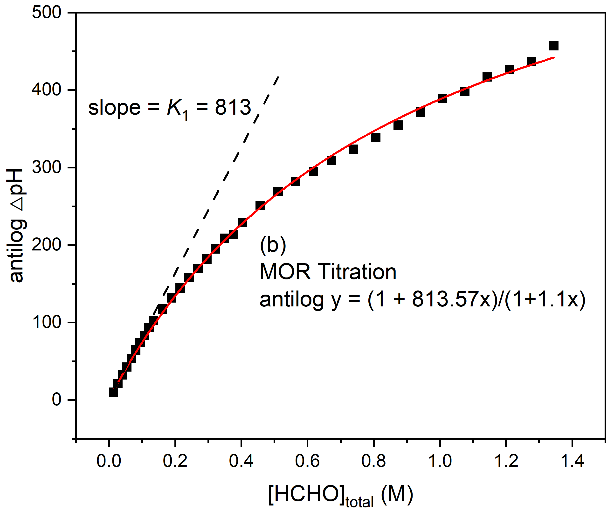


**Figure S4**. Titration of MOR with a formaldehyde solution. Measured change of (a) pH and (b) the antilog pH as a function of the formaldehyde concentrations at 24 ± 1 ℃. The symbols represent the experimental data and the lines represent fits of the results based on eq. S3. The dashed line in (b) is the predicted line based on the assumption without consideration of protonation of carbinolamine. Experimental conditions: [MOR]_0_ = 5 mM, 50% free base, [ionic strength] = 25 mM, [pH]_0_ = 8.56.


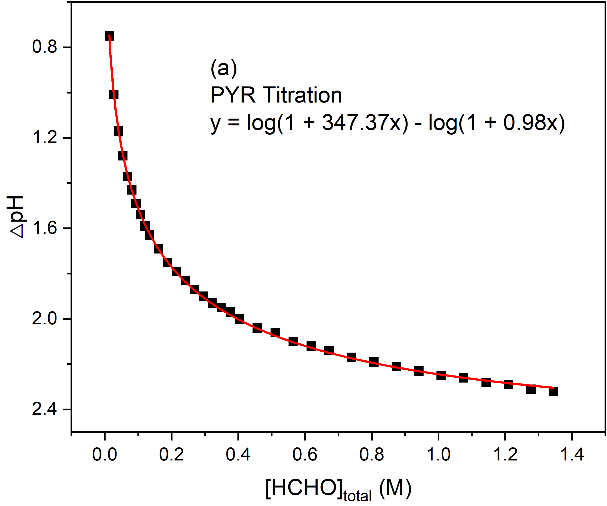

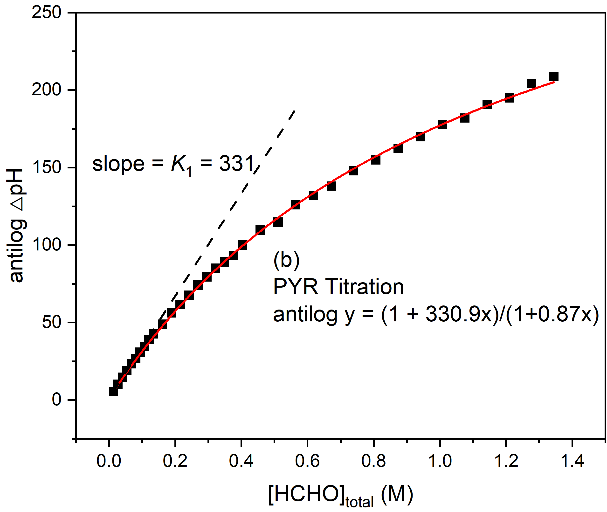


**Figure S5**. Titration of PYR with a formaldehyde solution. Measured change of (a) pH and (b) antilog pH as a function of the formaldehyde concentrations at 24 ± 1 ℃. The symbols represent the experimental data and the lines represent fits of the results based on eq. S3. The dashed line in (b) is the predicted line based on the assumption without consideration of protonation of carbinolamine. Experimental conditions: [PYR]_0_ = 5 mM, 50% free base, [ionic strength] = 25 mM, [pH]_0_ = 11.16.





**Figure S6.** Speciation of nitrite/nitrous acid as a function of pH.

**
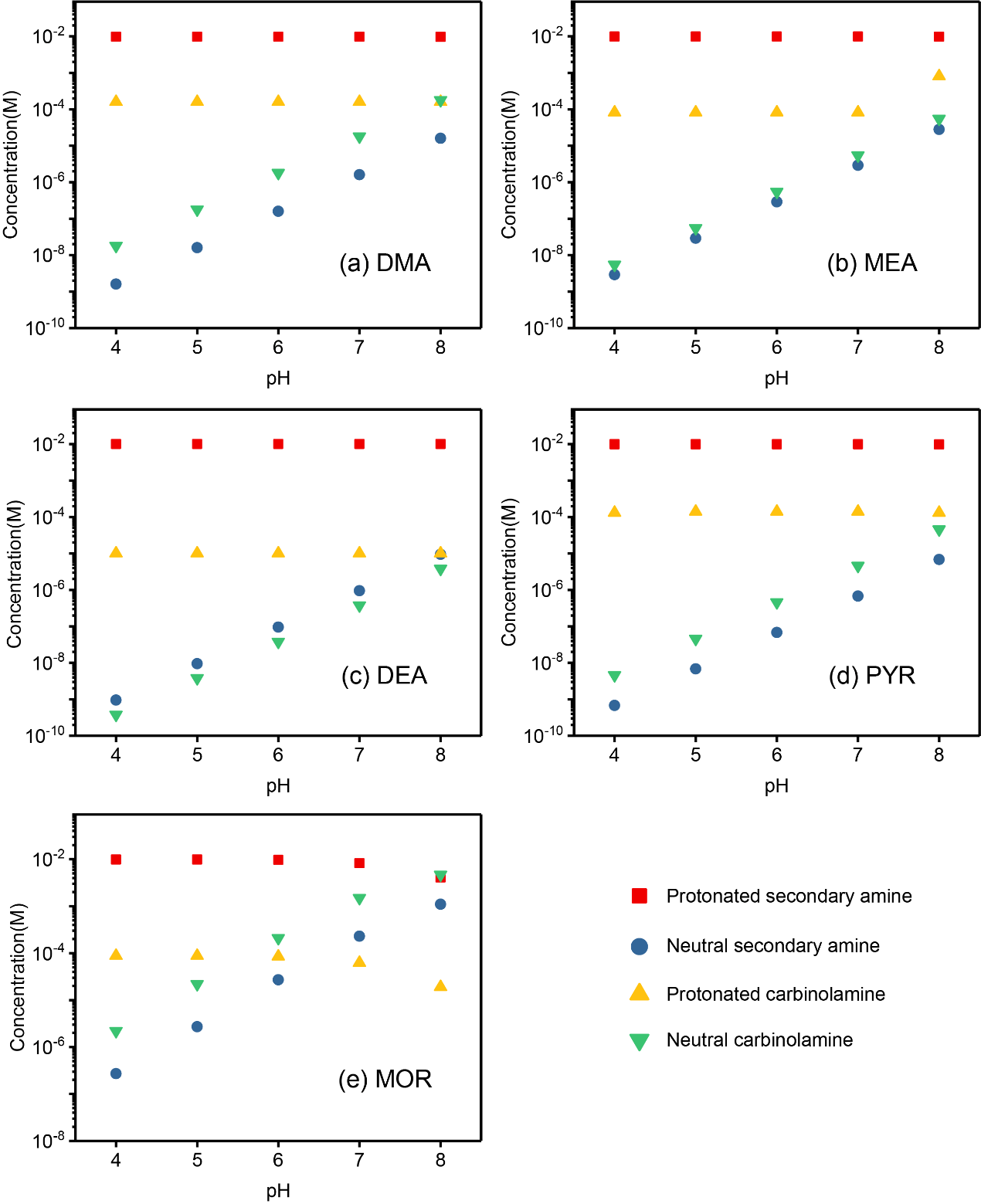
**

**Figure S7.** Calculated equilibrium speciation of secondary amines in presence of formaldehyde as a function of the pH: Concentrations of each species for the reaction of (a) DMA, (b) MEA, (c) DEA, (d) NPYR and (e) MOR with formaldehyde. Initial conditions: [DMA]_0_ = [MEA]_0_ = [DEA]_0_ = [PYR]_0_ = [MOR]_0_ = [HCHO]_0_ = 10 mM, pH = 4 – 8.












**Figure S8**. Experimental results for *N*-nitrosamine formation by nitrosation of secondary amines: Estimation of the contributions of secondary amine or iminium ion to the observed formation kinetics (*r*_obs_) for (a) NDMA, (b) NMEA, (c) NDEA and (d) NPYR formation at different pHs. Experimental conditions: [DMA]_0_ = [MEA]_0_ = [DEA]_0_ = [PYR]_0_ = [HCHO]_0_ = 10 mM, [NO_2_^-^]_0_ = 50 mM, pH = 4 - 8, phosphate buffer = 0.1 M.





**Figure S9**. Experimental results for *N*-nitrosamine formation by nitrosation of MOR: Estimation of the contributions of each pathway (secondary amine, iminium ion) to the observed formation kinetics (*r*_obs_) for NMOR formation at different pH values. Experimental conditions: [MOR]_0_ = [HCHO]_0_ = 10 mM, phosphate buffer = 0.1 M; [NO_2_^-^]_0_ = 5 mM (pH = 4), 12 mM (pH = 5) and 50 mM (pH = 6 - 8).





**Figure S10**. Predicted *r*_obs_ for the iminium ion pathway for NMOR formation at different pH values. Initial conditions: [MOR]_0_ = [HCHO]_0_ = 10 mM, [NO_2_^-^]_0_ = 50 mM, pH = 4 - 8, phosphate buffer = 0.1 M.












**Figure S11**. Experimental results of *N*-nitrosamine formation by nitrosation from the reactions of secondary amines with N_2_O_3_: Correlations between *r*_obs_ and [NO_2_^-^]^2^ for (a) NDMA, (b) NMEA, (c) NDEA and (d) NPYR formation in the absence of formaldehyde at pH= 4, 5, 6. Experimental conditions: [DMA]_0_ = [MEA]_0_ = [DEA]_0_ = [PYR]_0_ = 10 mM, [NO_2_^-^]_0_ = 20 - 50 mM, pH = 4 - 6, phosphate buffer = 0.1 M.

**



**

**Figure S12.** Experimental results of *N*-nitrosamine formation by nitrosation from the reaction of MOR with N_2_O_3_: Correlation between *r*_obs_ and [NO_2_^-^]^2^ for NMOR formation in absence of formaldehyde at different pH values. Experimental conditions: [MOR]_0_ = 10 mM, [NO_2_^-^]_0_ = 2 - 50 mM, pH = 4 - 7, phosphate buffer = 0.1 M.





**Figure S13.** Quantum chemically computed minimum energy pathway for the reaction of each protonated secondary amine with N_2_O_3_.





**Figure S14.** Quantum chemically computed minimum energy pathway for the reaction from protonated carbinolamine to the iminium ion.

**

**

**Figure S15.** Quantum chemically computed minimum energy pathway for the reaction of carbinolamine with nitrite.








**Figure S16.** Quantum chemically computed minimum energy pathway for the reactions of secondary amines with (a) methanediol and (b) formaldehyde.















**Figure S17.** Comparison of experimental and modeling results of the formation kinetics (*r*_obs_) and the contribution of various pathways for NDMA formation from DMA in the presence of formaldehyde at pH (a) 4, (b) 5, (c) 6, (d) 7, (e) 8. Experimental conditions: [DMA]_0_ = [HCHO]_0_ = 10 mM, [NO_2_^-^]_0_ = 1 - 50 mM, phosphate buffer = 0.1 M.

**



**

**



**

**Figure S18.** Comparison of experimental and modeling results for NDMA formation kinetics by the carbinolamine pathway (*r*_carbinolamine_) in presence of formaldehyde for different pHs: (a) pH 4, (b) pH 5, (c) pH 6 and (d) pH 7. Experimental conditions: [DMA]_0_ = [HCHO]_0_ = 10 mM, [NO_2_^-^]_0_ = 5 - 50 mM, phosphate buffer = 0.1 M.















**Figure S19.** Kinetic modeling (Table S1) for the evolution of carbinolamine ([(CH_3_)_2_NCH_2_OH]) concentrations during nitrosation in the presence of formaldehyde: (a) pH 4, (b) pH 5, (c) pH 6, (d) pH 7, (e) pH 8. Initial conditions: [DMA]_0_ = [HCHO]_0_ = 10 mM, [NO_2_^-^]_0_ = 50 mM, pH = 4 - 8.





**Figure S20.** Quantum chemically computed minimum energy pathways for the reactions of the carbonyl compounds-derived iminium ions with nitrite.











**Figure S21.** Modeling results of scenario 1 in Text S10 for NDMA formation from each pathway in the presence of formaldehyde: (a) pH 5, (b) pH 6, (c) pH 7, (d) pH 8. Experimental conditions: [DMA]_0_ = [HCHO]_0_ = 5 μM, [NO_2_^-^]_0_ = 6.5 × 10^-5^ M, pH = 5 - 8.











**Figure S22.** Modeling results of scenario 2 in Text S10 for NDMA formation from each pathway in the presence of formaldehyde: (a) pH 5, (b) pH 6, (c) pH 7, (d) pH 8. Experimental conditions: [DMA]_0_ = [HCHO]_0_ = 1 mM, [NO_2_^-^]_0_ = 1 mM, pH = 5 - 8.

**





**

**Figure S23.** Modeling results of scenario 3-1 in Text S10 for NDMA formation from each pathway in the presence of formaldehyde: (a) pH 5, (b) pH 7, (c) pH 9. Experimental conditions: [DMA]_0_ = [HCHO]_0_ = 1 mM, [NO_2_^-^]_0_ = 2.2 × 10^-7^ M, pH = 5 - 9.

**





**

**Figure S24.** Modeling results of scenario 3-2 in Text S10 for NDMA formation from each pathway in the presence of formaldehyde: (a) pH 5, (b) pH 7, (c) pH 9. Experimental conditions: [DMA]_0_ = [HCHO]_0_ = 0.1 M, [NO_2_^-^]_0_ = 2.2 × 10^-7^ M, pH = 5 - 9.

**





**

**Figure S25.** Modeling results of scenario 3-3 in Text S10 for NDMA formation from each pathway in the presence of formaldehyde: (a) pH 5, (b) pH 7, (c) pH 9. Experimental conditions: [DMA]_0_ = [HCHO]_0_ = 1 mM, [NO_2_^-^]_0_ = 0.1 M, pH = 5 - 9.

**Table S1.** Selected reactions for the modeling of NMDA and carbinolamine formation by nitrosation in the presence of formaldehyde.

| NO. | Reaction | Rate constant  (s^-1^ or M^-1^s^-1^) | Reference |
| --- | --- | --- | --- |
| 1 | HNO_2_ → H^+^ + NO_2_^-^ | 7.1 × 10^6^ | from p*K*a = 3.15,^17^ and *k*_2_ |
| 2 | H^+^ + NO_2_^-^ → HNO_2_ | 1.0 × 10^10^ | assumed^a^ |
| 3 | HNO_2_ + HNO_2_ → H_2_O + N_2_O_3_ | 17.7 | 18 |
| 4 | H_2_O + N_2_O_3_ → HNO_2_ + HNO_2_ | 5.9 × 10^3^ | from *K*a = 0.003,^17^ and *k*_3_ |
| 5 | (CH_3_)_2_NH + H^+^ →(CH_3_)_2_NH_2_^+^ | 1.0 × 10^10^ | assumed^a^ |
| 6 | (CH_3_)_2_NH_2_^+^ → (CH_3_)_2_NH + H^+^ | 1.7 × 10^-1^ | from p*K*a = 10.78,^1^ and *k*_5_ |
| 7 | (CH_3_)_2_NH + HCHO_total_^b^ → (CH_3_)_2_NCH_2_OH | 5.6 × 10^3^ | 19 |
| 8 | (CH_3_)_2_NCH_2_OH → (CH_3_)_2_NH + HCHO_total_^b^ | 4.9 | from *K* = 1150,^1^ and *k*_7_ |
| 9 | (CH_3_)_2_NH^+^CH_2_OH → (CH_3_)_2_NCH_2_OH + H^+^ | 1.1 × 10^2^ | from p*K*a=7.93 (this study), and *k*_10_ |
| 10 | (CH_3_)_2_NCH_2_OH + H^+^ → (CH_3_)_2_NH^+^CH_2_OH | 1.0 × 10^10^ | assumed^a^ |
| 11 | (CH_3_)_2_NH + N_2_O_3_ → (CH_3_)_2_NNO + other product | 1.2 × 10^8^ | this study |
| 12 | (CH_3_)_2_NCH_2_OH + N_2_O_3_ → (CH_3_)_2_NNO + other product | 1.7 × 10^8^ | this study |
| 13 | (CH_3_)_2_N^+^=CH_2_ + NO_2_^-^ → (CH_3_)_2_NNO + other product | 1.2 × 10^-4^ | this study |

^a^ The rate constants for protonation reactions were assumed to be 1.0 × 10^10^ M^-1^ s^-1^ to reach equilibrium instantaneously^19^.

^b^ Here, the formaldehyde concentration used in the kinetic modelling is the sum of formaldehyde and its hydrated form (methanediol) and the corresponding second-order rate constant is also an apparent second-order rate constant which takes both species into account.

**Table S2.** Calculated speciation for the reactions of the selected secondary amines with formaldehyde^a^ in the pH-range 4-8 based on eqs. S7 - S11 in Text S4.

| **pH** | **Secondary amine** | **R_2_NH_2_^+^**  **(M)** | **R_2_NH**  **(M)** | **R_2_NHCH_2_OH^+^**  **(M)** | **R_2_NCH_2_OH**  **(M)** | **HCHO_total_^b^**  **(M)** |
| --- | --- | --- | --- | --- | --- | --- |
| 8 | DMA | 9.7 × 10^-3^ | 1.6 × 10^-5^ | 1.6 × 10^-4^ | 1.8 × 10^-4^ | 9.7 × 10^-3^ |
|  | MEA | 9.8 × 10^-3^ | 2.8 × 10^-5^ | 8.1 × 10^-4^ | 5.5 × 10^-5^ | 9.9 × 10^-3^ |
|  | DEA | 1.0 × 10^-2^ | 9.5 × 10^-6^ | 1.0 × 10^-5^ | 3.8 × 10^-6^ | 1.0 × 10^-2^ |
|  | PYR | 9.8 × 10^-3^ | 6.8 × 10^-6^ | 1.3 × 10^-4^ | 4.6 × 10^-5^ | 9.8 × 10^-3^ |
|  | MOR | 4.1 × 10^-3^ | 1.1 × 10^-3^ | 1.9 × 10^-5^ | 4.7 × 10^-3^ | 5.2 × 10^-3^ |
| 7 | DMA | 9.8 × 10^-3^ | 1.6 × 10^-6^ | 1.6 × 10^-4^ | 1.8 × 10^-5^ | 9.8 × 10^-3^ |
|  | MEA | 9.9 × 10^-3^ | 2.9 × 10^-6^ | 8.2 × 10^-5^ | 5.5 × 10^-6^ | 9.9 × 10^-3^ |
|  | DEA | 1.0 × 10^-2^ | 9.5 × 10^-7^ | 1.0 × 10^-5^ | 3.8 × 10^-7^ | 1.0 × 10^-2^ |
|  | PYR | 9.9 × 10^-3^ | 6.8 × 10^-7^ | 1.4 × 10^-4^ | 4.6 × 10^-6^ | 9.9 × 10^-3^ |
|  | MOR | 8.2 × 10^-3^ | 2.3 × 10^-4^ | 6.2 × 10^-5^ | 1.5 × 10^-3^ | 8.4 × 10^-3^ |
| 6 | DMA | 9.8 × 10^-3^ | 1.6 × 10^-7^ | 1.6 × 10^-4^ | 1.8 × 10^-6^ | 9.8 × 10^-3^ |
|  | MEA | 9.9 × 10^-3^ | 2.9 × 10^-7^ | 8.2 × 10^-5^ | 5.5 × 10^-7^ | 9.9 × 10^-3^ |
|  | DEA | 1.0 × 10^-2^ | 9.5 × 10^-8^ | 1.0 × 10^-5^ | 3.8 × 10^-8^ | 1.0 × 10^-2^ |
|  | PYR | 9.9 × 10^-3^ | 6.8 × 10^-8^ | 1.4 × 10^-4^ | 4.6 × 10^-7^ | 9.9 × 10^-3^ |
|  | MOR | 9.7 × 10^-3^ | 2.7 × 10^-5^ | 8.5 × 10^-5^ | 2.1 × 10^-4^ | 9.7 × 10^-3^ |
| 5 | DMA | 9.8 × 10^-3^ | 1.6 × 10^-8^ | 1.6 × 10^-4^ | 1.8 × 10^-7^ | 9.8 × 10^-3^ |
|  | MEA | 9.9 × 10^-3^ | 2.9 × 10^-8^ | 8.2 × 10^-5^ | 5.5 × 10^-8^ | 9.9 × 10^-3^ |
|  | DEA | 1.0 × 10^-2^ | 9.5 × 10^-9^ | 1.0 × 10^-5^ | 3.8 × 10^-9^ | 1.0 × 10^-2^ |
|  | PYR | 9.9 × 10^-3^ | 6.8 × 10^-9^ | 1.4 × 10^-4^ | 4.6 × 10^-8^ | 9.9 × 10^-3^ |
|  | MOR | 9.9 × 10^-3^ | 2.7 × 10^-6^ | 8.8 × 10^-5^ | 2.2 × 10^-5^ | 9.9 × 10^-3^ |
| 4 | DMA | 9.8 × 10^-3^ | 1.6 × 10^-9^ | 1.6 × 10^-4^ | 1.8 × 10^-8^ | 9.8 × 10^-3^ |
|  | MEA | 9.9 × 10^-3^ | 2.9 × 10^-9^ | 8.2 × 10^-5^ | 5.5 × 10^-9^ | 9.9 × 10^-3^ |
|  | DEA | 1.0 × 10^-2^ | 9.5 × 10^-10^ | 1.0 × 10^-5^ | 3.8 × 10^-10^ | 1.0 × 10^-2^ |
|  | PYR | 9.9 × 10^-3^ | 6.8 × 10^-10^ | 1.3 × 10^-4^ | 4.6 × 10^-9^ | 9.9 × 10^-3^ |
|  | MOR | 9.9 × 10^-3^ | 2.7 × 10^-7^ | 8.8 × 10^-5^ | 2.2 × 10^-6^ | 1.0 × 10^-2^ |

^a^Conditions: [secondary amine]_0_ = [HCHO]_0_ = 0.01 M.

^b^Here, the concentration of formaldehyde is the sum of HCHO and CH_2_(OH)_2_. In aqueous solution, 99.9% formaldehyde exists in the hydrated form (CH_2_(OH)_2_).^2^

**Table S3.** Calculated speciation of different nitrogen species for varying nitrite concentrations and pH^a^ based on Text S2.

| **NaNO_2_**  **(M)** | **pH** | **HNO_2_**  **(M)** | **NO_2_^-^**  **(M)** | **N_2_O_3_**  **(M)** |
| --- | --- | --- | --- | --- |
| 0.05 | 8 | 7.1 × 10^-7^ | 5.0 × 10^-2^ | 1.5 × 10^-15^ |
|  | 7 | 7.1 × 10^-6^ | 5.0 × 10^-2^ | 1.5 × 10^-13^ |
|  | 6 | 7.1 × 10^-5^ | 5.0 × 10^-2^ | 1.5 × 10^-11^ |
|  | 5 | 7.0 × 10^-4^ | 4.9 × 10^-2^ | 1.5 × 10^-9^ |
|  | 4 | 6.2 × 10^-3^ | 4.4 × 10^-2^ | 1.2 × 10^-7^ |
| 0.03 | 8 | 4.2 × 10^-7^ | 3.0 × 10^-2^ | 5.4 × 10^-16^ |
|  | 7 | 4.2 × 10^-6^ | 3.0 × 10^-2^ | 5.4 × 10^-14^ |
|  | 6 | 4.2 × 10^-5^ | 3.0 × 10^-2^ | 5.4 × 10^-12^ |
|  | 5 | 4.2 × 10^-4^ | 3.0 × 10^-2^ | 5.2 × 10^-10^ |
|  | 4 | 3.7 × 10^-3^ | 2.6 × 10^-2^ | 4.1 × 10^-8^ |
| 0.01 | 8 | 1.4 × 10^-7^ | 1.0 × 10^-2^ | 6.0 × 10^-7^ |
|  | 7 | 1.4 × 10^-6^ | 1.0 × 10^-2^ | 6.0 × 10^-15^ |
|  | 6 | 1.4 × 10^-5^ | 1.0 × 10^-2^ | 6.0 × 10^-13^ |
|  | 5 | 1.4 × 10^-4^ | 9.9 × 10^-3^ | 5.8 × 10^-11^ |
|  | 4 | 1.2 × 10^-3^ | 8.8 × 10^-3^ | 4.6 × 10^-9^ |

^a^ Conditions: [nitrite] = 0.01 - 0.05 M, pH = 4 - 8 .

**Table S4.** Summary of determined second-order rate constants (*k*) for the reactions of secondary amines and carbinolamines with N_2_O_3_ and (*k*_iminium ion_×*K*_3_) for iminium ions at different pH.

| **Secondary amines** | **pH** | ***k*_R2NH_**  **(M^-1^ s^-1^)** | ***k*_carbinolaime_**  **(M^-1^ s^-1^)** | ***k*_iminium ion_**×***K*_3_**  **(M^-1^ s^-1^)** |
| --- | --- | --- | --- | --- |
| DMA | 8 | - | - | (1.16 ± 0.005) × 10^-4^ |
|  | 7 | - | - | - |
|  | 6 | (2.12 ± 0.03) × 10^8^ | (1.80 ± 0.003) × 10^8^ | - |
|  | 5 | (1.51 ± 0.12) × 10^8^ | (1.86 ± 0.02) × 10^8^ | - |
|  | 4 | (1.24 ± 0.09) × 10^8^ | (1.67 ± 0.03) × 10^8^ | - |
| MEA | 8 | - | - | (6.09 ± 0.004) × 10^-5^ |
|  | 7 | - | - | - |
|  | 6 | (3.27 ± 0.03) × 10^7^ | (8.11 ± 0.09) × 10^7^ | - |
|  | 5 | (3.39 ± 0.29) × 10^7^ | (8.48 ± 0.17) × 10^7^ | - |
|  | 4 | (3.02 ± 0.07) × 10^7^ | (7.90 ± 0.18) × 10^7^ | - |
| DEA | 8 | - | - | (4.20 ± 0.02) × 10^-5^ |
|  | 7 | - | - | - |
|  | 6 | (2.39 ± 0.06) × 10^7^ | (5.22 ± 0.39) × 10^7^ | - |
|  | 5 | (2.91 ± 0.14) × 10^7^ | (4.84 ± 0.33) × 10^7^ | - |
|  | 4 | (2.32 ± 0.10) × 10^7^ | (3.50 ± 0.15) × 10^7^ | - |
| PYR | 8 | - | - | (1.29 ± 0.07) × 10^-4^ |
|  | 7 | - | - | - |
|  | 6 | (7.49 ± 0.10) × 10^7^ | (1.25 ± 0.08) × 10^8^ | - |
|  | 5 | (6.58 ± 0.10) × 10^7^ | (1.26 ± 0.04) × 10^8^ | - |
|  | 4 | (6.81 ± 0.10) × 10^7^ | (1.07 ± 0.07) × 10^8^ | - |
| MOR | 8 | - | - | (3.26 ± 0.06) × 10^-3^ |
|  | 7 | (6.52 ± 0.11) × 10^7^ | - | - |
|  | 6 | (1.04 ± 0.01) × 10^8^ | - | - |
|  | 5 | (1.24 ± 0.005) × 10^8^ | - | - |
|  | 4 | (1.45 ± 0.05) × 10^8^ | - | - |

**Table S5.** Estimated second-order rate constants (*k*_iminium ion_) for the reactions of iminium ions and nitrite based on eq. S15 in Text S5^a, b^.

| Iminium ion | electrophilicity *E* | nucleophilicity *N* (nitrite)^20^ | *s_N_*^20^ | *k*  (M^-1^ s^-1^) |
| --- | --- | --- | --- | --- |
| 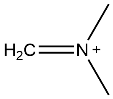 | -6.69^21^ | 17.2 | 0.72 | 7.59 × 10^7^ |
| 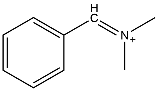 | -9.27^22^ | 17.2 | 0.72 | 5.13 × 10^5^ |
| 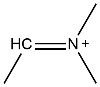 | Not available | 17.2 | 0.72 | Not available |
| 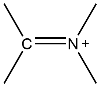 | Not available | 17.2 | 0.72 | Not available |

^a^ Rate constant were obtained in organic solvent (acetonitrile).

^b^ *k*_iminium ion_ values estimated by this method are only used for the section “*N*-nitrosamine Formation in the Presence of Other Carbonyl Compounds” in the main text, and are not comparable to those determined in Table S4. Because in Table S4, *k*_iminium ion_×*K*_3_ is used as an alternative to *k*_iminium ion_.

**Table S6.** Estimated p*K*_a_ values for MEA and carbinolamines by the Chemicalize software^23^.

| Name | Structure | p*K*_a_ |
| --- | --- | --- |
| MEA | **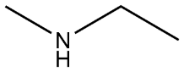** | 10.54 |
| (dimethylamino)methanol | 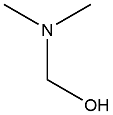 | 7.93 |
| (ethyl(methyl)amino)methanol | 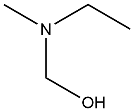 | 8.17 |
| (diethylamino)methanol | 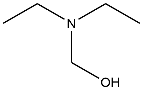 | 8.42 |
| pyrrolidin-1-ylmethanol | 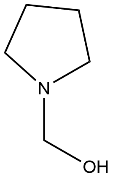 | 8.52 |
| morpholinomethanol | 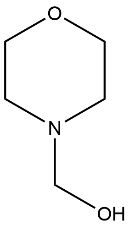 | 6.03 |

**Table S7:** Comparison of activation energies, enthalpies of activation, and Gibbs free energies of activation calculated at different levels of theory for the reactions of selected neutral secondary amines with N_2_O_3_. Enthalpies and Gibbs free energies were calculated at standard conditions of 298.15 K and 1 bar of ideal gas. Energy values in kcal/mol.

| Dimethylamine | | | |
| --- | --- | --- | --- |
| Level of theory | Δ*E*^act^_calc,aq_ | Δ*H*^act^_calc,aq_ | Δ*G*^act^_calc,aq_ |
| B3LYP/6-311+G(d,p) | -0.2 | 0.3 | 8.5 |
| M06-2X/Aug-cc-pVTZ//B3LYP/6-311+G(d,p) | -0.4 | 0.1 | 8.3 |
| CCSD(T)/Aug-cc-pVDZ//B3LYP/6-311+G(d,p) | -2.5 | -2.0 | 6.3 |
| Experiment |  | 0.2^15^ |  |
| Diethylamine | | | |
| Level of theory | Δ*E*^act^_calc,aq_ | Δ*H*^act^_calc,aq_ | Δ*G*^act^_calc,aq_ |
| B3LYP/6-311+G(d,p) | -1.0 | 0.0 | 8.8 |
| M06-2X/Aug-cc-pVTZ//B3LYP/6-311+G(d,p) | -1.4 | -0.4 | 8.4 |
| CCSD(T)/Aug-cc-pVDZ//B3LYP/6-311+G(d,p) | -4.2 | -3.3 | 5.6 |
| Experiment |  | 5.0^15^ |  |
| Morpholine | | | |
| Level of theory | Δ*E*^act^_calc,aq_ | Δ*H*^act^_calc,aq_ | Δ*G*^act^_calc,aq_ |
| B3LYP/6-311+G(d,p) | -0.1 | 0.4 | 9.4 |
| M06-2X/Aug-cc-pVTZ//B3LYP/6-311+G(d,p) | -0.6 | -0.1 | 9.0 |
| CCSD(T)/Aug-cc-pVDZ//B3LYP/6-311+G(d,p) | -3.3 | -2.8 | 6.3 |
| Experiment |  | 1.7^15^ |  |

**Table S8.** Summary of modeled concentrations of NDMA in a typical water treatment process (Scenario 1 in Text S10, for concentrations see below) as a function of pH in the presence and absence of formaldehyde.

| DMA^a^ | Nitrite^b^ | Formaldehyde^c^ | NDMA formation (ng/L)^d, e^ | | | |
| --- | --- | --- | --- | --- | --- | --- |
|  |  |  | pH 5 | pH 6 | pH 7 | pH 8 |
| 5 μM | 2.2 × 10^-7^ M | - | 1.9 × 10^-7^ | 1.9 × 10^-8^ | 1.9 × 10^-9^ | 1.9 × 10^-10^ |
| 5 μM | 2.2 × 10^-7^ M | 5 μM | 7.0 × 10^-6^ | 7.0 × 10^-6^ | 7.0 × 10^-6^ | 7.0 × 10^-6^ |
| 5 μM | 6.5 × 10^-5^ M | - | 1.7 × 10^-2^ | 1.7 × 10^-3^ | 1.7 × 10^-4^ | 1.7 × 10^-5^ |
| 5 μM | 6.5 × 10^-5^ M | 5 μM | 1.8 × 10^-2^ | 3.7 × 10^-3^ | 2.2 × 10^-3^ | 2.1 × 10^-3^ |

^a,c^DMA and formaldehyde concentrations were set at 5 μM to represent typical concentrations in a water treatment process^24^; ^b^ the nitrite concentration was set at 0.01mg/L (2.2 × 10^-7^ M) and 3mg/L (6.5 × 10^-5^ M) to represent a typically observed concentration and the WHO guideline limit for potable water, respectively^17^; ^d^NDMA formation potential reaction times were set at 24 h; ^e^pH values were set in the range of 5 - 8, which are common for water treatment processes^24^.

**Table S9.** Summary of modeled concentrations of NDMA in industrial discharges (Scenario 2 in Text S10, for concentrations see below) as a function of pH in the presence and absence of formaldehyde.

| DMA^a^ | Nitrite^b^ | Formaldehyde^c^ | NDMA formation (ng/L)^d, e^ | | | |
| --- | --- | --- | --- | --- | --- | --- |
|  |  |  | pH 5 | pH 6 | pH 7 | pH 8 |
| 1 mM | 1 mM | - | 7.9 × 10^2^ | 7.9 × 10^1^ | 7.9 × 10^0^ | 7.6 × 10^-1^ |
| 1 mM | 1 mM | 1 mM | 3.2 × 10^3^ | 1.5 × 10^3^ | 1.3 × 10^3^ | 1.3 × 10^3^ |

^a,c^DMA and formaldehyde concentrations were set at 1 mM to represent intermediate concentrations in industrial wastewater discharges^25, 26^; ^b^the nitrite concentration was set at 1 mM to represent an intermediate concentration detected in industrial wastewater discharges^27^; ^d^NDMA formation potential reaction times were set at 24 h; ^e^pH values were set in the range of 5 – 8 ^27^.

**Table S10.** Summary of modeled NDMA formation in industrial production processes (Scenario 3 in Text S10, for concentrations see below) as a function of pH in the presence and absence of formaldehyde.

| Scenario^a^ | DMA^b^ | Nitrite^c^ | Formaldehyde^d^ | NDMA formation (ng/L)^e, f^ | | |
| --- | --- | --- | --- | --- | --- | --- |
|  |  |  |  | pH 5 | pH 7 | pH 9 |
| 3-1 | 1 mM | 2.2 × 10^-7^ M | - | 3.8 × 10^-6^ | 3.8 × 10^-7^ | 3.7 × 10^-9^ |
|  | 1 mM | 2.2 × 10^-7^ M | 1 mM | 2.7 × 10^-1^ | 2.8 × 10^-1^ | 2.6 × 10^-1^ |
| 3-2 | 0.1 M | 2.2 × 10^-7^ M | - | 3.7 × 10^-3^ | 2.8 × 10^-5^ | 1.1 × 10^-8^ |
|  | 0.1 M | 2.2 × 10^-7^ M | 0.1 M | 2.0 × 10^3^ | 1.9 × 10^3^ | 6.6 × 10^2^ |
| 3-3 | 1 mM | 0.1 M | - | 7.3 × 10^6^ | 7.9 × 10^4^ | 5.8 × 10^2^ |
|  | 1 mM | 0.1 M | 1 mM | 1.5 × 10^7^ | 3.2 × 10^5^ | 1.2 × 10^5^ |

^a^Scenario 3-1: Trace nitrite with trace quantities of DMA and formaldehyde to model the situation when these reactants are present as impurities/intermediates during synthesis processes; Scenario 3-2: Trace nitrite with high concentrations of DMA and formaldehyde to model the situation when DMA and formaldehyde are used as reagents in a synthesis process and the water is polluted with traces of nitrite; Scenario 3-3: High nitrite concentration with trace concentrations of DMA and formaldehyde to model the presence of DMA and formaldehyde as impurities/intermediates during aqueous nitrosation reactions; ^b^DMA concentrations were set at 1mM and 1 M to represent low and high concentrations in industrial synthesis processes, respectively; ^c^nitrite concentrations were set at 0.01mg/L (2.2 × 10^-7^ M) and 1 M to represent an impurity and a reagent in industrial synthesis processes, respectively; ^d^formaldehyde concentrations were set at 1mM and 1 M to represent low and high concentrations in industrial synthesis processes, respectively; ^e^NDMA formation potential reaction time was set at 24 h; ^f^pH values were set in the range of 3 - 9 to represent different industrial production processes.

**References**

1. Kallen, R. G.; Jencks, W. P., Equilibria for the Reaction of Amines with Formaldehyde and Protons in Aqueous Solution. *J. Biol. Chem.* **1966,** *241*, (24), 5864-5878.

2. Keeper, L. K.; Roller, P. P., *N*-Nitrosation by Nitrite Ion in Neutral and Basic Medium. *Science* **1973,** *181*, (4106), 1245.

3. Casado, J.; Leis, J. R.; Mosquera, M.; Paz, L. C.; Peña, M. E., Kinetic Studies on the Formation of Nitrosamines 5.pdf. *Monatshefte für Chemie / Chemical Monthly* **1981,** *127*, 179-192.

4. Eldin, S.; Digits, J. A.; Huang, S.-T.; Jencks, W. P., Lifetime of an Aliphatic Iminium Ion in Aqueous Solution. *J. Am. Chem. Soc.* **1995,** *117*, (24), 6631-6632.

5. Mayr, H.; Patz, M., Scales of Nucleophilicity and Electrophilicity: A System for Ordering Polar Organic and Organometallic Reactions. *Angew. Chem. Int. Ed. Engl.* **1994,** *33*, (9), 938-957.

6. Mayr, H., Reply to T. W. Bentley: Limitations of the s(E+N) and Related Equations. *Angew. Chem. Int. Ed.* **2011,** *50*, (16), 3612-3618.

7. Becke, A. D., Density‐functional thermochemistry. III. The role of exact exchange. *J. Chem. Phys.* **1993,** *98*, (7), 5648-5652.

8. Lee, C.; Yang, W.; Parr, R. G., Development of the Colle-Salvetti correlation-energy formula into a functional of the electron density. *Phys. Rev. B* **1988,** *37*, (2), 785-789.

9. Vosko, S. H.; Wilk, L.; Nusair, M., Accurate spin-dependent electron liquid correlation energies for local spin density calculations: a critical analysis. *Can. J. Phys.* **1980,** *58*, (8), 1200-1211.

10. Marenich, A. V.; Cramer, C. J.; Truhlar, D. G., Universal Solvation Model Based on Solute Electron Density and on a Continuum Model of the Solvent Defined by the Bulk Dielectric Constant and Atomic Surface Tensions. *J. Phys. Chem. B* **2009,** *113*, (18), 6378-6396.

11. Zhao, Y.; Truhlar, D. G., The M06 suite of density functionals for main group thermochemistry, thermochemical kinetics, noncovalent interactions, excited states, and transition elements: two new functionals and systematic testing of four M06-class functionals and 12 other functionals. *Theor. Chem. Acc.* **2008,** *120*, (1), 215-241.

12. Pople, J. A.; Head‐Gordon, M.; Raghavachari, K., Quadratic configuration interaction. A general technique for determining electron correlation energies. *J. Chem. Phys.* **1987,** *87*, (10), 5968-5975.

13. Pari, S.; Wang, I. A.; Liu, H.; Wong, B. M., Sulfate radical oxidation of aromatic contaminants: a detailed assessment of density functional theory and high-level quantum chemical methods. *Environ. Sci. Process Impacts* **2017,** *19*, (3), 395-404.

14. Trogolo, D.; Arey, J. S.; Tentscher, P. R., Gas-Phase Ozone Reactions with a Structurally Diverse Set of Molecules: Barrier Heights and Reaction Energies Evaluated by Coupled Cluster and Density Functional Theory Calculations. *J. Phys. Chem. A* **2019,** *123*, (2), 517-536.

15. Casado, J.; Castro, A.; Leis, J. R.; López Quintela, M. A.; Mosquera, M., Kinetic studies on the formation of *N*-nitroso compounds VI. The reactivity of N_2_O_3_ as a nitrosating agent. *Monatshefte für Chemie / Chemical Monthly* **1983,** *114*, (6), 639-646.

16. Ridd, J. H., Diffusion Control and Pre-association in Nitrosation, Nitration and Halogenation. In *Adv. Phys. Org. Chem.*, Gold, V.; Bethell, D., Eds. Academic Press: 1978; Vol. 16, pp 1-49.

17. Ashworth, I. W.; Dirat, O.; Teasdale, A.; Whiting, M., Potential for the Formation of *N*-Nitrosamines during the Manufacture of Active Pharmaceutical Ingredients: An Assessment of the Risk Posed by Trace Nitrite in Water. *Org. Process Res. Dev.* **2020,** *24*, (9), 1629-1646.

18. Butler, A. R.; Ridd, J. H., Formation of nitric oxide from nitrous acid in ischemic tissue and skin. *Nitric Oxide* **2004,** *10*, (1), 20-24.

19. Das, S.; Schuchmann, M. N.; Schuchmann, H.-P.; Sonntag, C. V., The production of the superoxide radical anion by the OH radical-induced oxidation of trimethylamine in oxygenated aqueous solution. The kinetics of the hydrolysis of (hydroxymethyl)dimethylamine. *Chem. Ber.* **1987,** *120*, (3), 319-323.

20. Tishkov, A. A.; Schmidhammer, U.; Roth, S.; Riedle, E.; Mayr, H., Ambident Reactivity of the Nitrite Ion Revisited. *Angew. Chem. Int. Ed.* **2005,** *44*, (29), 4623-4626.

21. Mayr, H.; Kempf, B.; Ofial, A. R., π-Nucleophilicity in Carbon−Carbon Bond-Forming Reactions. *Acc. Chem. Res.* **2003,** *36*, (1), 66-77.

22. Appel, R.; Chelli, S.; Tokuyasu, T.; Troshin, K.; Mayr, H., Electrophilicities of Benzaldehyde-Derived Iminium Ions: Quantification of the Electrophilic Activation of Aldehydes by Iminium Formation. *J. Am. Chem. Soc.* **2013,** *135*, (17), 6579-6587.

23. ChemAxon Chemicalize. <https://chemaxon.com/products/chemicalize>

24. Marron, E. L.; Prasse, C.; Buren, J. V.; Sedlak, D. L., Formation and Fate of Carbonyls in Potable Water Reuse Systems. *Environ. Sci. Technol.* **2020,** *54*, (17), 10895-10903.

25. Lotfy, H. R.; Rashed, I. G., A method for treating wastewater containing formaldehyde. *Water Res.* **2002,** *36*, (3), 633-637.

26. Chang, Q.-l. J. A. M. R., Treatment of Industrial Dimethylamine Wastewater in a Distillation Tower at Lab Scale. **2013,** *634-638*, 326 - 329.

27. Zeng, T.; Glover, C. M.; Marti, E. J.; Woods-Chabane, G. C.; Karanfil, T.; Mitch, W. A.; Dickenson, E. R., Relative Importance of Different Water Categories as Sources of *N*-Nitrosamine Precursors. *Environ. Sci. Technol.* **2016,** *50*, (24), 13239-13248.
